# Penalized landmark supermodels (penLM) for dynamic prediction for time-to-event outcomes in high-dimensional data

**DOI:** 10.1186/s12874-024-02418-9

**Published:** 2025-01-27

**Authors:** Anya H. Fries, Eunji Choi, Summer S. Han

**Affiliations:** 1 Department of Management Science and Engineering, Stanford University, Stanford, CA 94304 USA; 2 Quantitative Sciences Unit, Department of Medicine, Stanford University School of Medicine, 3180 Porter Drive, Office 118, Stanford, CA 94304 USA; 3 Department of Population Health Sciences, Weill Cornell Medicine, New York, NY 10065 USA; 4 Department of Neurosurgery, Stanford University School of Medicine, Stanford, CA 94304 USA

**Keywords:** Dynamic prediction, Landmark, Penalization, Prediction accuracy, Longitudinal data, Competing risks, Cancer survivors, Survival data

## Abstract

**Background:**

To effectively monitor long-term outcomes among cancer patients, it is critical to accurately assess patients’ dynamic prognosis, which often involves utilizing multiple data sources (e.g., tumor registries, treatment histories, and patient-reported outcomes). However, challenges arise in selecting features to predict patient outcomes from high-dimensional data, aligning longitudinal measurements from multiple sources, and evaluating dynamic model performance.

**Methods:**

We provide a framework for dynamic risk prediction using the penalized landmark supermodel (penLM) and develop novel metrics ($\:\overline{AUC}_{w}\:$ and $\:\overline{BS}_{w}\:$) to evaluate and summarize model performance across different timepoints. Through simulations, we assess the coverage of the proposed metrics’ confidence intervals under various scenarios. We applied penLM to predict the updated 5-year risk of lung cancer mortality at diagnosis and for subsequent years by combining data from SEER registries (2007–2018), Medicare claims (2007–2018), Medicare Health Outcome Survey (2006–2018), and U.S. Census (1990–2010).

**Results:**

The simulations confirmed valid coverage (~ 95%) of the confidence intervals of the proposed summary metrics. Of 4,670 lung cancer patients, 41.5% died from lung cancer. Using penLM, the key features to predict lung cancer mortality included long-term lung cancer treatments, minority races, regions with low education attainment or racial segregation, and various patient-reported outcomes beyond cancer staging and tumor characteristics. When evaluated using the proposed metrics, the penLM model developed using multi-source data ($\:\overline{AUC}_{w}\:$of 0.77 [95% confidence interval: 0.74–0.79]) outperformed those developed using single-source data ($\:\overline{AUC}_{w}\:$range: 0.50–0.74).

**Conclusions:**

The proposed penLM framework with novel evaluation metrics offers effective dynamic risk prediction when leveraging high-dimensional multi-source longitudinal data.

**Supplementary Information:**

The online version contains supplementary material available at 10.1186/s12874-024-02418-9.

## Background

With recent advances in cancer treatment and early detection [1], effectively monitoring long-term outcomes has become more important among cancer patients. In particular, assessing patients’ dynamic prognosis is critical as it can incorporate a patient’s evolving health status or external stimulus (e.g., changing treatment) to guide effective clinical decision-making [2, 3]. This approach contrasts with traditional methods, which predict the risk of patients’ outcome (e.g., mortality) at a fixed time (e.g., diagnosis) using patients’ baseline information [4, 5]. Evaluating long-term outcomes often involves utilizing multiple data sources, such as tumor registries and patient-reported outcomes, to obtain further insight into effective monitoring [6–8]. However, challenges arise when data collection methods and assessment times differ across data sources. Dynamic prediction models, like the landmark supermodel [2, 3, 9], have the potential to address these challenges while providing updated risk estimates.

Concurrently, selecting predictive features from complex, high-dimensional data is challenging in dynamic risk prediction due to computational burden and model interpretability. Traditional feature selection techniques like stepwise- or domain knowledge-based selection [10, 11] become infeasible with increasing dimensionality. Joint models [12, 13], an alternative method for dynamic prediction, become intractable with numerous time-varying covariates as model complexity grows quickly [14], leading to challenges in numerical stability. Machine learning methods like random forests [15] and deep learning [16] can handle high-dimensionality but compromise on explainability. Other statistical learning approaches [17–19] tackle variable selection in survival data, but focus on noisy data rather than updated predictions or competing risks. Additionally, using a compound covariate [20] or a regularized latent class model [21], can reduce dimensions, but assessing a variable’s association with disease progression is challenging. Thus, there is a critical need for advanced methodologies in dynamic risk prediction for high-dimensional data that maintain interpretability and computational efficiency.

The remaining key challenge includes effectively evaluating and comparing the performance of various dynamic models over different time points. Traditional evaluation metrics like time-dependent AUC or Brier Score [22] assess and compare model performance at a specific time (landmark). However, currently, no metrics exist that can combine several time-dependent measures spanning different landmarks into a single score, together with a sufficient confidence interval or hypothesis testing framework. While simultaneous confidence bands proposed by Blanche et al. [22] could be relevant, difficulties remain in model comparison as different models may be superior at different landmarks [23], and multiple hypothesis testing must be accounted for. Importantly, alternative methods are not model-agnostic; for example, the dynamic discrimination index [24] is only suitable for joint models and information criteria metrics depend on model-specific likelihood constructions.

In this study, we develop a penalized landmark supermodel (penLM) framework for dynamic prediction in high-dimensional data, which can help manage large data while retaining interpretability and controlling overfitting. We also propose a novel model-agnostic evaluation metric that summarizes model performance across different time points into a single score. We apply the proposed methods to predict the 5-year lung cancer (LC) mortality among LC patients using multiple data sources and compare the proposed penLM with an alternative dynamic modeling framework.

## Methods

### General framework for the landmark supermodel

The landmark supermodel closely resembles the standard Cox [25] and cause-specific Cox (CSC) models for competing risks [26]. Further details are described in our prior work [27] and Supplemental Methods 1 of the Additional file 1. One way to conceptualize the supermodel is as a smooth aggregation of Cox (CSC) models over pre-defined time points (“landmarks”), denoted $\:\left\{{s}_{0},\dots\:,{s}_{L}\right\}$, which is used to perform updated $\:w$-year risk prediction. From a landmark time $\:s\in\:\left[{s}_{0},{s}_{L}\right],$ the hazard for $\:j\:$th event (“cause”)$\:\:\:(j=1,\:2,\dots\:,\:J)$ at time $\:t\:(s\le\:t\le\:s+w)$is:

$$\:{h}_{j}\left(t|Z\left(s\right),s\right)={h}_{j0}\left(t\right)\text{exp}\left({\alpha\:}_{j}\left(s\right)+\:{\beta\:}_{j}{\left(s\right)}^{T}Z\left(s\right)\right),$$

where $\:Z\left(s\right)$ are the most up-to-date values of an individual’s covariates at time $\:s$ and $\:{\alpha\:}_{j}\left(s\right)$ models the main effects of the landmark time. The interaction of $\:s$ with the covariates, modeled by $\:{\beta\:}_{j}\left(s\right)$, captures the time-dependent effects of covariates. For example, $\:\beta\:\left(s\right)=\:{\beta\:}_{0}+\:{\beta\:}_{1}s$ models a main and linear time-dependent effect.

**Fig. 1:**
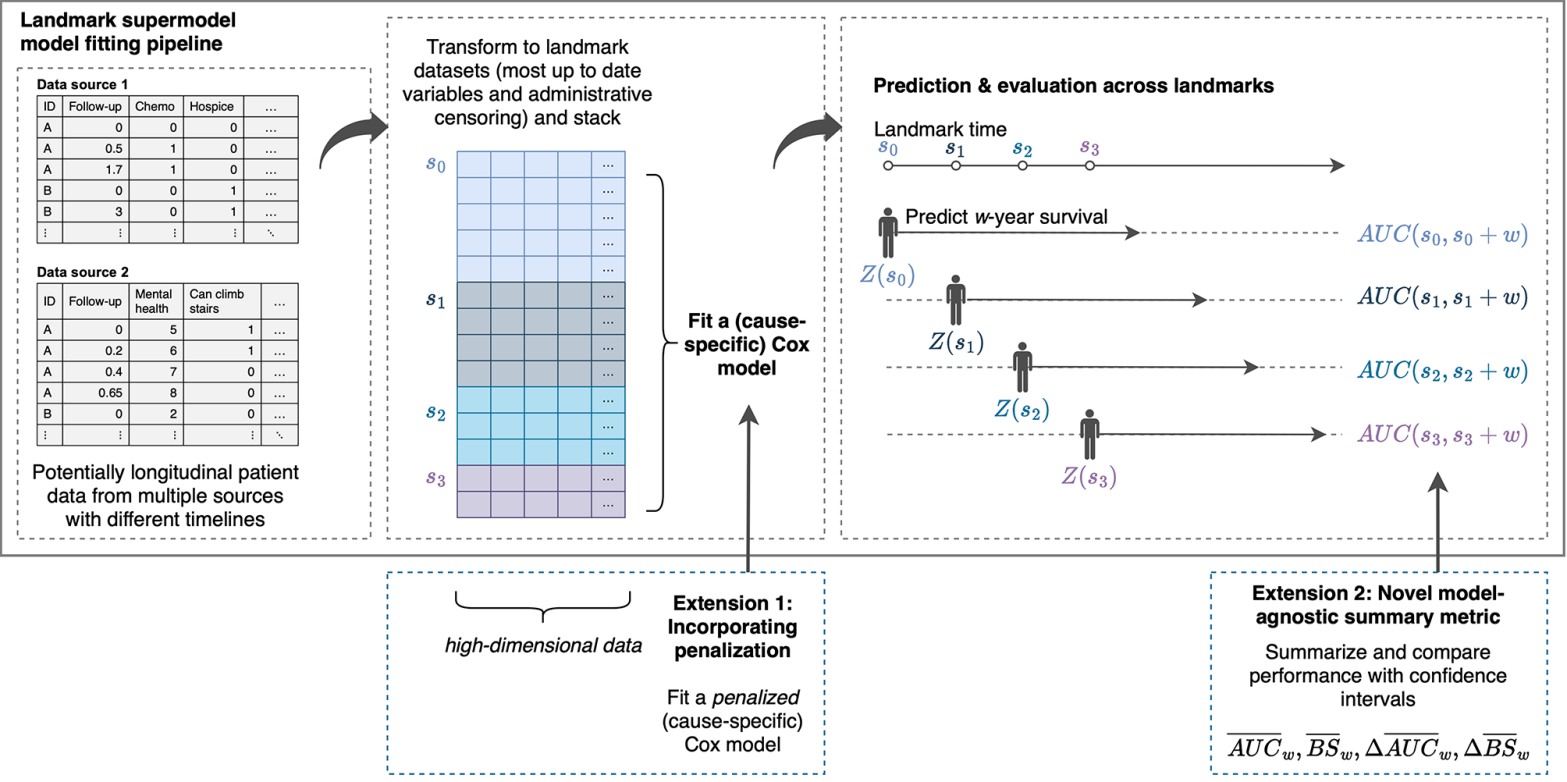
Landmark supermodel model-fitting pipeline. This framework facilitates dynamic risk prediction by first selecting key time points known as “landmarks.” Longitudinal patient data are then transformed into distinct datasets for each landmark using the most up-to-date variables of patients still under observation, subject to administrative censoring. These datasets are stacked into an extended dataset, on which a (cause-specific) Cox model is fitted and termed a “supermodel.” This model forms the basis for making and evaluating dynamic risk predictions at each landmark. The framework is extended in two ways: firstly, by introducing penalization to the supermodel to better handle high-dimensional data and prevent overfitting; secondly, by proposing a model-agnostic summary metric that averages performance measures (AUC, Brier Score) across landmarks, allowing for an overview of the model’s predictive ability over time with corresponding confidence intervals

The model fitting process is illustrated in Fig. 1. After selecting landmarks {$\:{s}_{0},\dots\:,\:{s}_{L}\}$ and a risk prediction window $\:w$, the study cohort is transformed into distinct datasets for each landmark $\:s,$ using the most up-to-date variables of individuals still under observation and applying censoring at $\:s+w$. These datasets are combined into a stacked dataset, on which a Cox (CSC) model is fit (i.e., supermodel).

### Extending the landmark supermodel with penalization: penLM

To deal with a large dataset with many parameters and time-dependent effects, we propose extending our prior work [7] by introducing penalization (Fig. 1). Specifically, a model is fit by maximizing the penalized log pseudo-partial likelihood of the supermodel. For a single-cause model, this is:

$$\begin{aligned}\:&\text{log}\:ip{l}^{*}\left(\beta,\alpha\right)-\lambda\:p\left(\beta,\alpha\right)  :=  \\ &\qquad\sum\limits_{i=1}^{n}\text{log}\left(\prod\limits_{\left\{s:\:s\le {T}_{i}\le s+w\right\}}{\left(\frac{{\exp}\left({Z}_{i}{\left(s\right)}^{T}\beta\left(s\right)+\alpha\left(s\right)\right)}{\sum\limits_{\left\{s:\:s\le {T}_{i}\le s+w\right\}}\sum\limits_{j\in R\left({T}_{i}\right)}{\exp}\left({Z}_{j}{\left(s\right)}^{T}\beta\left(s\right)+\alpha\left(s\right)\right)}\right)}^{{\eta}_{i}}\:\right)-\lambda\:p(\beta,\alpha)\end{aligned} $$

where $\:n$ is the number of patients, $\:R\left(T\right)$ is the risk set of patients alive at $\:T,\:\:\:$ and $\:{\eta\:}_{i},\:{T}_{i}\:$ are the occurrence of an event and time-to-event for patient $\:i$, respectively. The penalty $\:p\left(\cdot\:\right)$ can be a LASSO (the L1-norm), Ridge (the L2-norm), or an elastic net (a combination of the two) [28–30].

### Evaluating dynamic predictions: traditional approach

While there is no gold standard for comparing dynamic models, traditional time-dependent AUC and Brier Score [22] are typically considered. These metrics evaluate models at a specific time point, $\:s$ (a landmark). Time-dependent AUC, i.e. $\:AUC\left(s,s+w\right),$ evaluates the model’s discriminative ability to predict the event of interest occurring during $\:[s,\:s+w]$. Similarly, Brier Score, i.e. $\:BS\left(s,s+w\right),$ evaluates the model’s predictive accuracy by estimating the average squared difference between the observed vs. predicted risk at $\:[s,\:s+w]$ (Supplemental Methods 2.1.-2.2. in the Additional file 1). The time-dependent metrics are often evaluated multiple times across various landmarks, which can make it challenging to effectively compare different models, as relative performance may vary across times.

### Proposed summary metrics for dynamic model evaluation

We propose new metrics that summarize and compare the performance of various models (Fig. 1) by combining a set of time-dependent metrics across landmarks. To address correlation between time-dependent metrics—as the same individuals are assessed at multiple landmarks—we extended Blanche et al.’s [22] asymptotic i.i.d. decomposition to the multivariate case in building a confidence interval (CI), thus avoiding resampling and computational burden. Notably, the proposed metrics are entirely model-agnostic (i.e., not limited to a particular model), enabling comparisons across different modeling frameworks and even for misspecified models.

The proposed metrics that combine the time-dependent metrics from a set of landmarks $\:s\in\:\mathcal{S}$ are as follows:

$$\:\overline{\theta}_{w}=\frac{1}{\left|\mathcal{S}\right|}\:\sum\limits_{s\in\:\mathcal{S}}\theta\:(s,s+w)\:,$$

where $\:\theta\:(s,s+w)$ =$\:AUC\left(s,s+w\right)$ (for discrimination) or $\:\theta\:(s,s+w)$ =$\:BS\left(s,s+w\right)$ (for predictive accuracy). We estimate the CI interval of this metric using Lemma 1 (Supplemental Methods 2.3, Additional file 1), valid under independent censoring and for testing data. This method can additionally provide a P-value to test the difference in performance between models, $\:{\mathcal{H}}_{0}:{\Delta\:}{\overline{AUC}}_{w}=0$ and $\:{\mathcal{H}}_{0}:{\Delta\:}{\overline{BS}}_{w}=0$.

The proposed methods are available in our R package, dynamicLM, on GitHub.

### Simulation studies

We conducted simulations to evaluate the new summary metric’s finite sample behavior [31, 32]. We estimated the coverage probability of the summary metrics (i.e., the probability that the CI contains the true value of the summary metric), which is expected around 95% for a 95% CI. For the model comparison tests, we assessed power and type I error.

To evaluate coverage probability, we simulated 500 test and training datasets using a simplified landmark model [33] (Supplemental Methods 3.1, Additional file 1) under varying sample sizes (*n* = 500,750,1500), landmark intervals (LMI = 2,4,6) and censoring rates (0%, 15%, 30%, 50%) (Supplemental Methods 3.2 and Supplementary Table S2, Additional file 1). The landmark model included three fixed (i.e., non-time-varying) variables and one longitudinal variable (i.e., time-varying variable whose value can change over time) generated at three landmarks. A landmark supermodel was then fitted to each training dataset, and predictions were made on test data (Supplemental Methods 3.2, Additional file 1). We calculated time-dependent AUCs and Brier scores at each landmark, which were used to estimate the summary metrics ($\:{\overline{BS}}_{w}$ and $\:{\overline{AUC}}_{w}$) and their CIs. We then counted the proportion of CIs that included the true values across different scenarios (Supplemental Methods 3.2 and Supplementary Table S2, Additional file 1).

To evaluate the type I error of the proposed tests, we fitted two equivalent models, $\:{M}_{1}$ and $\:{M}_{1}^{*}$, to training data (Supplemental Methods 3.3, Additional file 1). Using predictions on testing data, we estimated the proposed metrics, $\:{\overline{BS}}_{w}$ and $\:{\overline{AUC}}_{w}$ for each model. We then tested the performance difference between $\:{M}_{1}$ and $\:{M}_{1}^{*}$ ($\:{\mathcal{H}}_{0}:{\Delta\:}{\overline{AUC}}_{w}=0$ and $\:{\mathcal{H}}_{0}:{\Delta\:}{\overline{BS}}_{w}=0$) across various significance levels. Type I error was calculated across over 500 simulations with a sample size of 3000. When assessing power, we compared model $\:{M}_{1}$ with a different model, $\:{M}_{2}$ (Supplemental Methods 3.4, Additional file 1). Without the proposed metric, performance comparison relies on testing traditional time-dependent metric tests [22] (i.e., testing $\:{\mathcal{H}}_{0}:{\Delta\:}AUC\left(s,\:s+w\right)=0$ and $\:{\mathcal{H}}_{0}:{\Delta\:}BS\left(s,\:s+w\right)=0\:$ at each landmark $\:s\in\:\{{s}_{1},\:\dots\:,\:{s}_{k}\}$), adjusted for multiple testing performed across *k* landmarks (Supplemental Methods 3.4, Additional file 1). We compared the power of the proposed tests vs. the traditional time-dependent tests under varying sample size (*n*=500, 750, 1500), varying number of landmarks at which longitudinal data was generated (*k*=3, 5, 7) and censoring rates (0%, 15%, 30%, 50%) to observe their effects on power.

### Application: LC mortality prediction using integrated data sources

#### Primary outcome, study cohort, and data integration

We applied the proposed penLM framework and summary metrics to evaluate the dynamic risk of LC-specific mortality among LC patients. In particular, we predicted the five-year risk of LC mortality at diagnosis (i.e., baseline) and one- and two-years post-diagnosis using their updated patient data. As LC patients who are heavy smokers are susceptible to other-cause deaths (a “competing risk”), we used a cause-specific Cox (CSC) supermodel in the penLM framework.

**Fig. 2:**
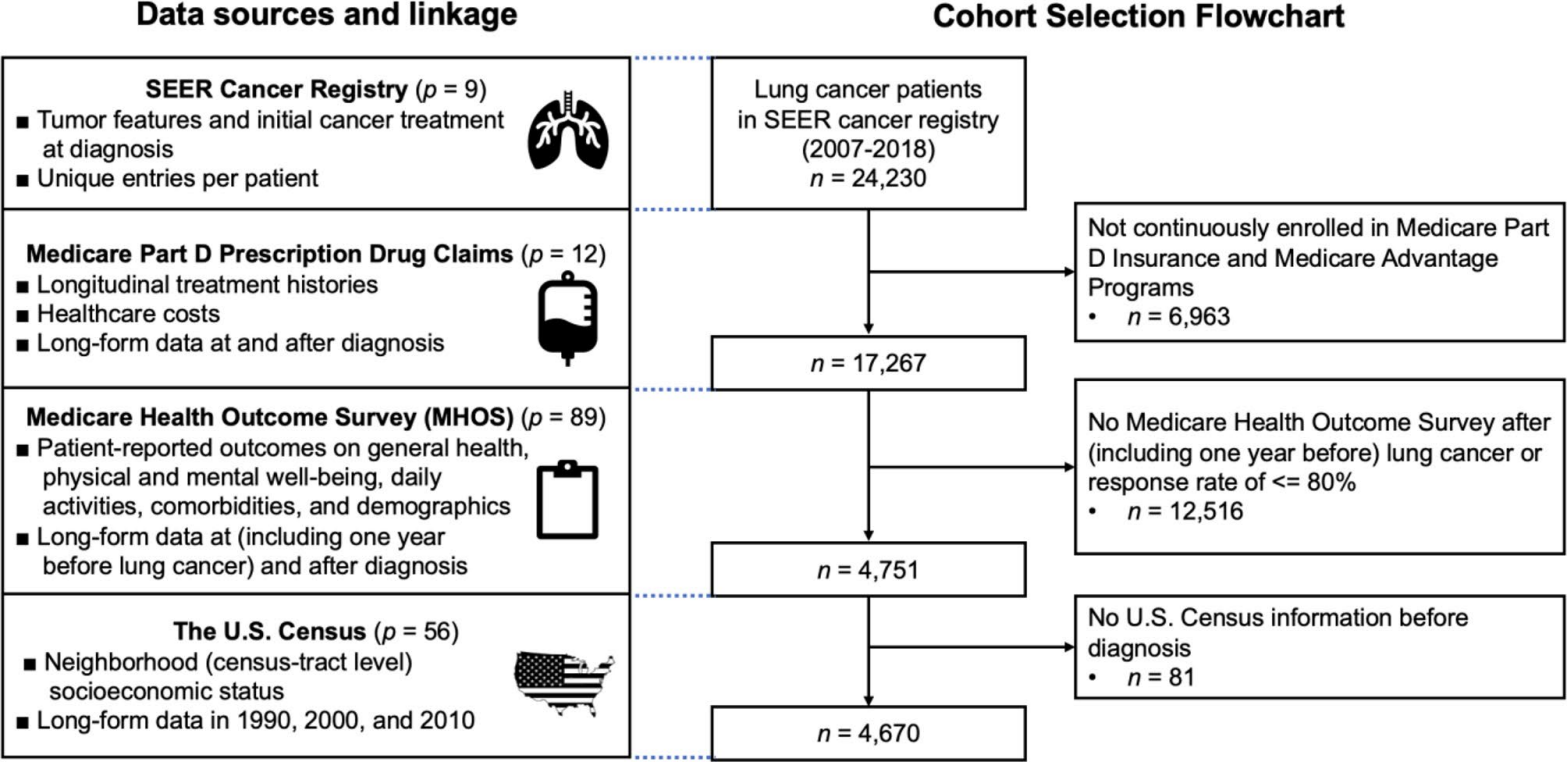
Study cohort selection for lung cancer mortality analysis using SEER-Medicare/MHOS/US Census data. The following shows the flowchart of multiple data processing and study cohort selection. Abbreviations: *n* = number of unique individuals, p = number of covariates

The study cohort (Fig. 2) comprises 4,670 patients diagnosed with LC between 2007 and 2017 in the SEER who were continuously enrolled in Medicare Part D and Medicare Advantage programs and completed at least one patient-reported outcome survey (Supplemental Methods 4, Additional file 1).

This study used both static and longitudinal data sources (Fig. 2). The static U.S. SEER cancer registry (2007–2018) provides tumor characteristics and cancer treatment information at diagnosis. The longitudinal data include the Medicare Health Outcome Survey (MHOS, 2006–2018) [34], containing biennial patient-reported outcomes from Medicare Advantage enrollees, Medicare Part D Insurance Claims (2007–2018), documenting prescription drug purchases, and the U.S. Census, which combines the 1990 and 2000 Censuses and the 2008–2012 American Community Survey to provide neighborhood-level socio-economic data. The penLM framework integrates data from multiple sources into a stacked dataset by aligning the study baseline (i.e., LC diagnosis) and carrying the most recent covariate information forward (Supplemental Methods 4, Additional file 1).

### PenLM modeling and model comparisons

We fit the proposed penLM supermodel to this data, using quarterly landmarks from 0 to 2 years, a 5-year prediction window, and a ridge penalty. This penalty handles the high level of correlation between our coefficients and their time-dependent effects [28], shrinking less important coefficients. We used 70% of data for model development and 30% for validation.

We compared penLM with an alternative dynamic risk prediction method: disjoint CSC models (Supplemental Methods 4, Additional file 1). We assessed model discrimination and predictive accuracy on the validation set using two sets of metrics: (i) traditional time-dependent AUC and Brier Score at *s* = 0,1,2 years post-diagnosis and (ii) using the proposed summary metrics for discrimination (i.e., $\:{\overline{AUC}}_{w=5}$) and predictive accuracy ($\:{\overline{BS}}_{w=5}$) that average the traditional time-dependent metrics into a single score.

To evaluate the utility of multi-source data in dynamic LC-specific mortality prediction, we compared the performance of the penLM model fitted using multi-source data against penLM models using individual sources.

## Results

### Simulation results

The simulation results show that the proposed summary metrics achieve overall good coverage (Table S3). Across all scenarios, the mean coverage probabilities for the summary Brier score ($\:{\overline{BS}}_{w}$) and summary AUC ($\:{\overline{AUC}}_{w})\:$were 93.7% and 94.7%, respectively, indicating that the CIs of the proposed metrics correctly include the true $\:{\overline{BS}}_{w}$ ($\:{\overline{AUC}}_{w}$) around 94% (95%) of the time—close to the 95% expected at a 0.05 level. The results of traditional time-dependent metrics are in Table S4.

**Fig. 3:**
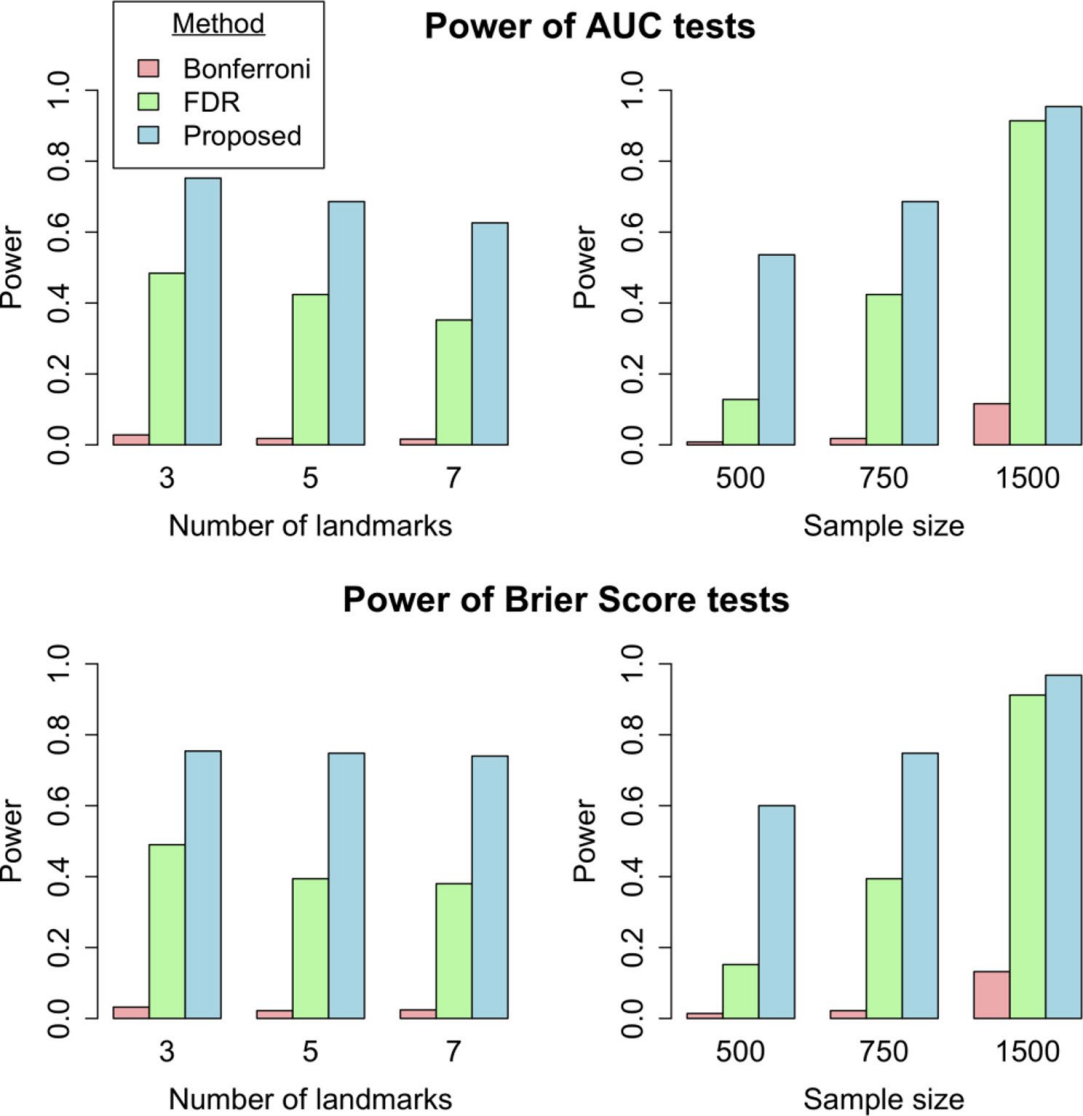
Simulation results of the power of the comparison tests based on the proposed summary metrics. The power of the proposed summary AUC (denoted $\:{\overline{AUC}}_{w}$) and Brier Score (denoted $\:{\overline{BS}}_{w}$) is shown for the tests $\:{\mathcal{H}}_{0}:{\Delta\:}{\overline{AUC}}_{w}=0$ and $\:{\mathcal{H}}_{0}:{\Delta\:}{\overline{BS}}_{w}=0\:\:$under varying number of landmarks (*k*={3,5,7}; first column) and sample size (*n*={500,750,1500}; second column) for a 5% significance level. Without the proposed metric, performance comparison relies on testing a set of *k* traditional, landmark-specific time-dependent metrics, adjusted for multiple testing (see Supplemental Methods 3.4. of the Additional file 1), for which we used the Bonferroni method and the Benjamini-Hochberg method to control for a false discovery rate (FDR) of 5%. The data simulation method is further described in Supplemental Methods 3.4. of the Additional file 1. The two figures in the first column show power for varying numbers of landmarks with a fixed sample size of *n*=750 for $\:{\overline{AUC}}_{w}$ and $\:{\overline{BS}}_{w}$, respectively. The two figures in the second column show power over varying sample sizes with 5 landmarks for $\:{\overline{AUC}}_{w}$ and $\:{\overline{BS}}_{w}$, respectively. A full panel of results for all combinations of sample sizes and landmarks is shown in Figure S2. Abbreviations: FDR: false discovery rate, *k*: number of landmarks, *n*: sample size

The simulation study also demonstrated that the model comparison tests based on the proposed summary AUC and Brier Score metrics have correct type I errors (Table S5), which are only slightly affected by censoring. Further, the power simulation results in Fig. 3 showed that the proposed tests have higher power than the traditional methods (i.e., a set of landmark-specific time-dependent tests adjusted for multiple testing) across various scenarios. The difference is most pronounced for small sample sizes. Figure S2A-C contains additional results for power simulations, including under censoring. While power generally decreases slightly under higher censoring rates, the proposed summary metrics still exhibit increased power over traditional methods.

### Application for predicting LC mortality

The study cohort included 4,670 LC patients with 166 covariates (Fig. 2), mostly female (52.2%) and White (75.4%) (Table S1). Of these patients, 41.5% died from LC, 40.1% died from other causes (competing events), and 18.3% were censored. Patients who died from LC were more likely to have a distant stage (60.0%) and small cell LC histology (15.8%). They lived in areas with a comparatively higher Hispanic population and lower education levels and had poorer baseline mental (6.78/8), physical (4.49/8), and social functioning (6.17/8) scores, where higher scores indicate better functioning.

**Fig. 4:**
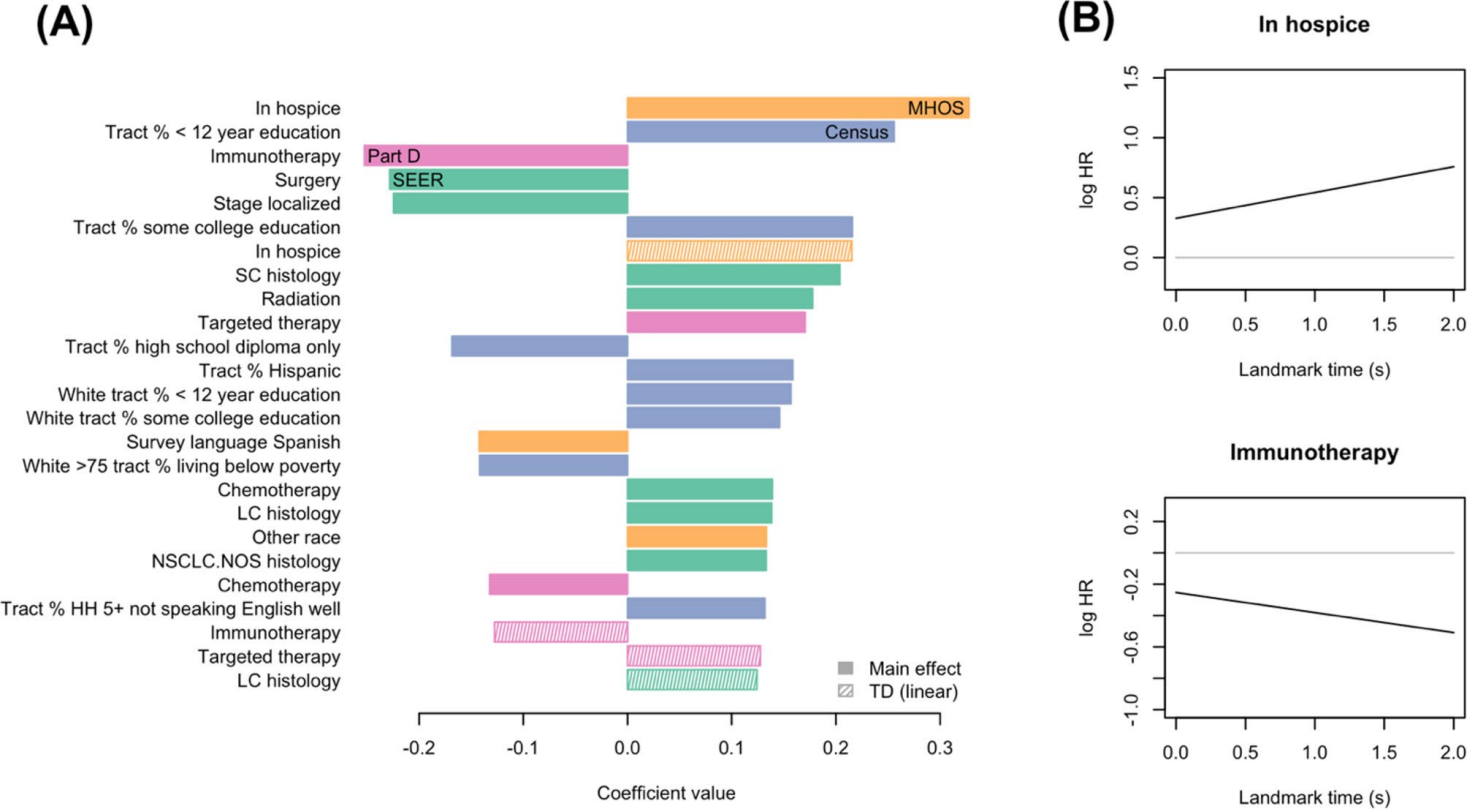
Top predictive factors for lung cancer-specific mortality identified through penLM using SEER-Medicare/MHOS/US Census data. The (**A**) panel shows the top 25 predictors with the largest model coefficients of the final fitted model using the proposed penLM supermodel. The data sources of the coefficients are given by the color (SEER: green, MHOS: orange, US Census: blue, Medicare Part D: purple), and shading indicates the main effects (solid) and time-dependent (“TD” linear) effects (i.e., interaction between the main effect and time/landmark) (lined). The (**B**) panel shows the time-dependent hazard ratios of the two covariates with the largest linear time-dependent effect observed in this analysis. Abbreviations: TD: time-dependent, logHR: log hazard ratio, MHOS: Medicare Health Outcomes Survey, SC: small cell, LC: large cell, NSCLC.NOS: non-small call lung cancer not otherwise specified, HH 5 + = households age 5+

The penLM supermodel using integrated multi-source data (SEER-Medicare, MHOS, U.S. Census, and Medicare Part D) identified several key predictors for LC-specific mortality (Fig. 4A), which included tumor characteristics-related variables in SEER, e.g., localized stage at diagnosis (baseline hazard ratio [HR] 0.80) and first-course surgery (HR 0.80) that were associated with reduced LC mortality. In Medicare Part D claims data, immunotherapy (HR 0.78) and chemotherapy (HR 0.88) were associated with reduced LC mortality (with time-dependent effects; Fig. 4B), whereas targeted therapy was associated with increased mortality (HR 1.19). MHOS highlighted the importance of comorbidities (such as hospice status [HR 1.39]) as well as patient-reported activities of daily living (such as “hearing most things people say” [HR 0.92]). U.S. Census data indicated higher LC mortality in areas with lower education (HR 1.29), larger Hispanic populations (HR 1.17), and limited English proficiency (HR 1.14).

**Fig. 5:**
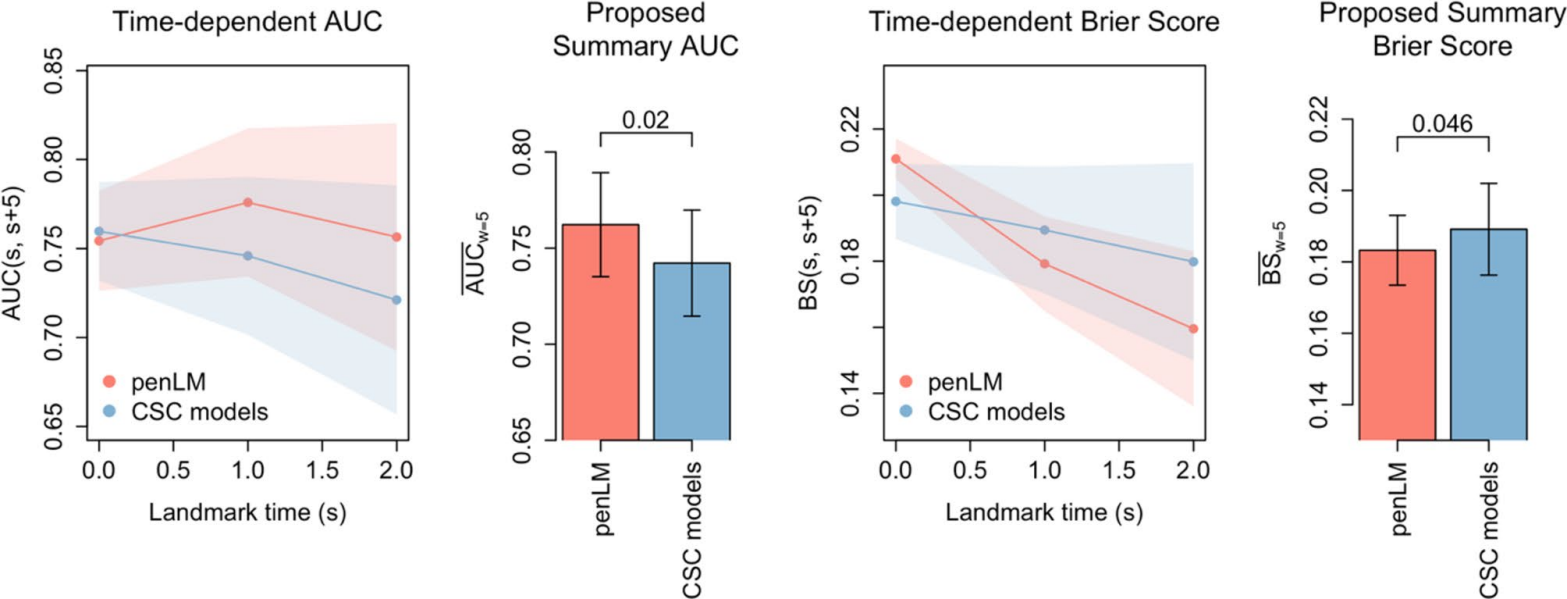
Comparison of model performance for predicting lung cancer mortality: proposed penLM model vs. CSC models. Performance of the proposed penalized landmark (penLM) supermodel (in red) compared to landmark-specific cause-specific Cox models (CSC models, in blue) is evaluated on held-out test data (*n* = 1401) from SEER-Medicare/MHOS/US Census. The first panel (or the third panel) shows the performance of each prediction model measured by the traditional time-dependent $\:AUC(s,\:s+w)$ (or Brier Score, $\:BS(s,\:s+w)$) that were assessed three times, i.e., at a landmark time of *s* = 0, 1, and 2 years from the initial diagnosis, for a prediction window of w=5 years from each landmark, with 95% confidence intervals shaded. The Brier score metric can take a value between 0 and 1; a lower value means higher accuracy. The second panel (or the fourth panel) shows the performance of each model measured by the proposed metric, $\:{\overline{AUC}}_{w=5}$ (or $\:{\overline{BS}}_{w=5}$) that summarizes the set of correlated time-dependent $\:AUC(s,\:s+w)$ (or time-dependent $\:BS(s,\:s+w)$) over three landmarks, with a 95% confidence interval added to each bar. Abbreviations: BS = Brier Score, penLM: penalized landmark supermodel, CSC: cause-specific Cox

We compared the performance of the penLM model vs. an alternative approach using landmark-specific CSC models (Fig. 5) built on multi-source data. Given that the nature of the CSC models is a set of disjoint models as each is fit at one landmark using the subset of patients alive (Supplemental Methods 4, Additional file 1), its application led to unsmooth time-dependent effects over landmarks (Figure S3). We performed model comparison by applying the traditional time-dependent metrics and found that the two models’ relative performance varied over landmarks. For example, the CSC models performed slightly better at baseline, but penLM showed superior performance, thereafter. Thus, summarizing relative model performance was challenging. When we applied the proposed metrics, the penLM model exhibited superior performance both in discrimination ($\:{\overline{AUC}}_{w=5}$ of 0.76 vs. 0.74 for penLM vs. CSC; P-value=0.020) and predictive accuracy ($\:{\overline{BS}}_{w=5}$ 0.18 vs. 0.19 for penLM vs. CSC; *P* = 0.046).

**Fig. 6:**
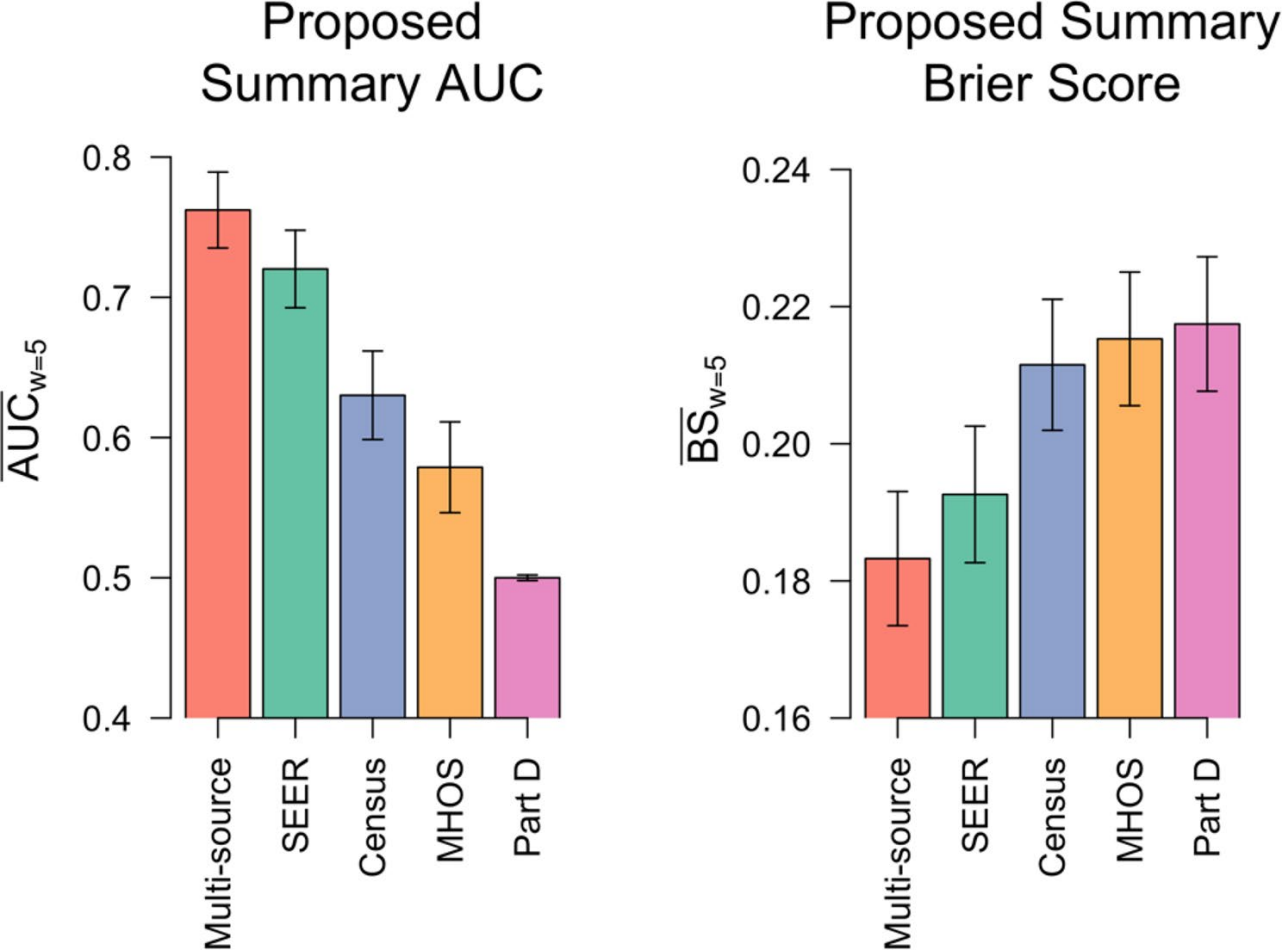
Comparison of penLM model performance for predicting lung cancer mortality: multiple vs. individual data sources. Performance of the proposed penLM that integrates all data sources (“Multi-source” in red, i.e., SEER, Medicare Part D, MHOS, and US Census) using test data (*n* = 1401). This model is compared to the four models that were developed using individual source data: (i) SEER in green, (ii) MHOS in orange, (iii) US Census in purple, and (iv) Medicare Part D in pink. The first panel shows the discriminatory performance of each model measured by the proposed metric, $\:{\overline{AUC}}_{w=5}$, that summarizes the set of correlated time-dependent $\:AUC(s,\:s+w)$ over time for a prediction window of w=5 years. The second panel shows the predictive accuracy of each model measured by the proposed metric, $\:{\overline{BS}}_{w=5}$, that summarizes the set of correlated time-dependent $\:BS(s,\:s+w)$ over time for a prediction window of w = 5 years. All model pairs had significant (adjusted P-value < 0.05) performance differences from each other, where the P-values were determined using comparison tests of our proposed summary metric, $\:{\Delta\:}{\overline{AUC}}_{w=5}$ and $\:{{\Delta\:}\overline{BS}}_{w=5}$, and adjusted to control for a false discovery rate of 5% using the Benjamini-Hochberg method. Abbreviations: MHOS = Medicare Health Outcomes Survey Data, BS = Brier Score. Note: 95% confidence intervals have been added to each bar

To evaluate the impact of using multiple data sources in predicting LC mortality, we compared the performance of the penLM model using multiple data sources (Fig. 4) vs. the models developed using individual data sources (Fig. 6). The results showed that the multi-source penLM model achieves higher discrimination ($\:{\overline{AUC}}_{w=5}$=0.76 [95% CI, 0.74–0.79]) than the single-source models ($\:{\overline{AUC}}_{w=5}$ range: 0.50–0.72). Similarly, predictive accuracy, measured by $\:{\overline{BS}}_{w=5}$, was also superior (i.e., lowest) for the multi-source model. Among the single-source models, the model based on SEER performed best, while the predictive performance of the U.S. Census-based model and the MHOS survey-based model improved over time (Figure S3).

## Discussion

In this study, we introduced a novel framework for dynamic risk prediction for time-to-event outcomes, incorporating competing events, by penalizing the landmark supermodel with various penalties. This framework is tailored for variable selection in high-dimensional data and facilitates the integration of multiple data sources to provide updated prognoses as patients’ conditions evolve. The proposed summary metrics streamlined model assessment and comparison, enabling effective model evaluation. Our simulation showed that the proposed metrics had good coverage probability with improved power to test differences in predictive model performance vs. traditional time-dependent metrics. The proposed framework and metrics are implemented in a freely available R package, dynamicLM.

Applying the proposed framework to LC data demonstrated that integrating multiple data sources enhances the predictive performance for long-term cancer outcomes vs. the models developed based on individual data sources. The single-source model using MHOS survey data showed increasing predictive accuracy over time, emphasizing the importance of continuously surveilling patient-reported outcomes. This approach underscores the complexity of LC progression and the need for multidisciplinary strategies in cancer care and surveillance.

Several prior studies provided dynamic risk modeling approaches for survival outcomes, but most were primarily designed for low-dimensional data. While one recent study [35] applied penalization to a variation of the landmark supermodel (without competing events), their prediction did not align with the landmark approach, as their baseline hazard did not change across landmarks, potentially biasing risk estimates. In contrast, penLM, tailored for high-dimensional data, varies the baseline hazards across landmarks and handles various time-to-event outcomes, including competing events. This further contrasts with the alternative framework (landmark-specific CSC models), which required manual variable selection, impractical for large datasets like genomic data or machine-learning features. The proposed penLM outperformed the CSC models’ performance while addressing these limitations by offering functional benefits like penalization, interpretability, and a streamlined modeling process.

Effectively modeling time-dependent effects in survival data offers further insights into how the effects of predictors on study outcomes change over time, which also helps address the inevitable violation of the proportional hazard assumption [36]. One key strength of the penLM is its flexibility in incorporating the time-dependent effects of predictors by allowing smooth effects on model outcomes over landmarks by utilizing a stacked supermodel. In contrast, the landmark-specific CSC models result in unsmooth time-dependent effects over landmarks, which may partly explain their diminishing performance in predicting LC mortality over time in our application.

The present study has limitations. As with all penalized methods, our approach does not provide confidence intervals (and hence P-values) for model coefficients. Thus, traditional statistical inference, such as hypothesis testing, may not be straightforward, posing challenges in interpreting the significance of individual predictors. While penLM is suitable for high-dimensional datasets, the standard landmark supermodel and other alternatives like joint modeling may be well-suited for risk prediction with a well-defined number of covariates.

Both the CSC landmark supermodel and proposed summary metrics assume independent censoring, which is appropriate for many situations but may be too limiting for some cases. The inference procedures of the time-dependent and proposed summary metrics also require independent test data to ensure that the individuals form an i.i.d. sample with independent risk scores across subjects. This limits the utility of these methods to hold out test data. Under violation of either of these assumptions–independent censoring or the use of independent test data–the statistical properties (e.g., coverage rates) of the proposed summary metrics are not expected to hold. Additional simulations could further assess the robustness of the summary metrics: either exploring the violation of these assumptions or the finite sample results of alternative models (e.g., joint models, penalized landmark supermodels). Lastly, the generalizability of the LC mortality prediction in this study may require external validation to confirm the applicability of our findings across various populations.

## Conclusions

To conclude, we developed a penalized landmark supermodel (penLM) framework as a potentially powerful tool for dynamic risk prediction in high-dimensional settings that can integrate multiple data sources, enabling further insights for personalized medicine and clinical decision-making. Applications of the proposed penLM and novel metrics in predicting LC mortality show that the model that utilizes multi-source data (vs. single-source) improves predictive performance, highlighting the value of comprehensive data integration in evaluating long-term cancer outcomes. The proposed novel, model-agnostic evaluation metrics facilitate effective dynamic risk model comparisons by summarizing performance across time with confidence intervals and offering a hypothesis test for model comparison. These advancements offer enhanced adaptability and accuracy in analyzing an extensive range of longitudinal and baseline covariates, thereby driving progress in epidemiologic and clinical research.

## Electronic supplementary material

Below is the link to the electronic supplementary material.

Supplementary Material 1

## Data Availability

The data underlying this article are available through application to the National Cancer Institute Division of Cancer Control and Population Sciences (https://healthcaredelivery.cancer.gov/seer-mhos/overview/).

## Abbreviations

AUC: Area Under the Curve
BS: Brier Score
CI: Confidence Interval
CSC: Cause-Specific Cox
HR: Hazard Ratio
i.i.d.: Independent and Identically Distributed
LC: Lung Cancer
LASSO: Least Absolute Shrinkage and Selection Operator
MHOS: Medicare Health Outcome Survey
penLM: Penalized Landmark Supermodel
SEER: Surveillance, Epidemiology, and End Results
