# Supplementary material for: Penalized landmark supermodels (penLM) for dynamic prediction for time-to-event outcomes in high-dimensional data
Source: BMC Med Res Methodol. 2025 Jan 27;25:22. doi: 10.1186/s12874-024-02418-9 (PMC11771018; doi:10.1186/s12874-024-02418-9)
Supplement: Supplementary file 1 — Supplementary Material 1 [file 12874_2024_2418_MOESM1_ESM.docx]

# Supplementary Materials

CONTENTS

1. **Supplementary Tables**

Table S1. Baseline patient characteristics of the study cohort for lung cancer mortality analysis

Table S2. Simulation scenariosfor evaluating the coverage probability of the proposedsummary metrics

Table S3. Simulation results of evaluating the coverage probability of the proposed summary metrics

Table S4. Simulation results of the coverage probability of the traditional time-dependent AUC and time-dependent Brier Score

Table S5. Simulation results of the type I error of the comparison test based on the proposed summary metrics

1. **Supplementary Figures**

Figure S1. Illustrating coverage rates: confidence intervals of the proposed summary AUC across 100 simulations

Figure S2. Simulation results of the power of the proposed summary performance tests

1. Results over varying number of landmarks
2. Results over varying sample size
3. Results over varying censoring rates

Figure S3. Examples of time-dependent effects (coefficient trajectories over time) of three variables using the proposed penLM and alternative (CSC models) for lung cancer mortality analysis.

1. **Supplementary Methods**
2. The landmark supermodel
   1. Overview
   2. Landmarking
   3. Sliding landmark model and extension to competing risks
   4. The landmark supermodel
   5. Prediction
   6. Penalization (penLM)
3. Evaluating model performance
   1. Traditional metrics
   2. Model performance: Estimators and inference for traditional metrics
   3. Model performance: novel summary metrics
4. Simulation methods
   1. Simulating a landmark dataset
   2. Evaluating coverage probability
   3. Evaluating Type I error of the proposed performance comparison test
   4. Evaluating the power of the proposed performance comparison test
5. Methods for lung cancer mortality prediction using integrated data sources
6. **References**

# Supplementary Tables

**Table S1: Baseline patient characteristics of the study cohort for lung cancer mortality analysis.** Values are reported in the number of patients [*n*] (percentage of patients [%]) unless otherwise specified (with *) as the mean value (standard deviation) for patients.

|  |  | | Event | | | | | |
| --- | --- | --- | --- | --- | --- | --- | --- | --- |
|  | Overall | | Censored | | Lung cancer Mortality | | Other cause mortality | |
|  | *n* | *%* | *n* | *%* | *n* | *%* | *n* | *%* |
| Number of individuals | 4670 |  | 855 |  | 1942 |  | 1873 |  |
| Time-to-event* | 1.91 | (2.29) | 4.46 | (2.78) | 1.09 | (1.38) | 1.58 | (1.93) |
| ***Basic demographic and MHOS variables*** | | | | | | | | |
| Male | 2231 | (47.8) | 331 | (38.7) | 980 | (50.5) | 920 | (49.1) |
| Race |  |  |  |  |  |  |  |  |
| Black | 535 | (11.5) | 92 | (10.8) | 234 | (12) | 209 | (11.2) |
| Hispanic | 315 | (6.7) | 69 | (8.1) | 145 | (7.5) | 101 | (5.4) |
| Other | 301 | (6.4) | 59 | (6.9) | 166 | (8.6) | 76 | (4.1) |
| White | 3519 | (75.4) | 635 | (74.3) | 1397 | (71.9) | 1487 | (79.4) |
| Mental health scale* | 6.91 | (2.3) | 7.28 | (2.13) | 6.78 | (2.38) | 6.88 | (2.26) |
| Physical functioning scale* | 4.65 | (3.16) | 5.38 | (3.11) | 4.49 | (3.19) | 4.49 | (3.11) |
| Social functioning scale* | 6.41 | (3.2) | 7.07 | (3.03) | 6.17 | (3.29) | 6.35 | (3.15) |
| ***SEER cancer registry variables*** | | | | | | | | |
| Stage |  |  |  |  |  |  |  |  |
| distant | 2223 | (47.6) | 107 | (12.5) | 1166 | (60) | 950 | (50.7) |
| localized | 1283 | (27.5) | 475 | (55.6) | 358 | (18.4) | 450 | (24) |
| regional | 1164 | (24.9) | 273 | (31.9) | 418 | (21.5) | 473 | (25.3) |
| Histology |  |  |  |  |  |  |  |  |
| AD | 1842 | (39.4) | 475 | (55.6) | 682 | (35.1) | 685 | (36.6) |
| LC and NSCLC.NOS | 429 | (9.2) | 33 | (3.9) | 207 | (10.7) | 189 | (10.1) |
| OTH | 746 | (16) | 97 | (11.3) | 299 | (15.4) | 350 | (18.7) |
| SC | 528 | (11.3) | 24 | (2.8) | 306 | (15.8) | 198 | (10.6) |
| SQ | 1125 | (24.1) | 226 | (26.4) | 448 | (23.1) | 451 | (24.1) |
| Chemotherapy | 1464 | (31.3) | 218 | (25.5) | 799 | (41.1) | 447 | (23.9) |
| Radiation | 1120 | (24) | 176 | (20.6) | 730 | (37.6) | 214 | (11.4) |
| Surgery | 968 | (20.7) | 434 | (50.8) | 211 | (10.9) | 323 | (17.2) |
| ***U.S. Census variables*** |  |  |  |  |  |  |  |  |
| Tract % age 25+ with <12-year education* | 16.39 | (11.5) | 14.35 | (10.61) | 17.58 | (12.01) | 16.08 | (11.21) |
| Tract % age 25+ with a high school diploma only* | 29.21 | (9.34) | 28.46 | (9.67) | 28.83 | (9.53) | 29.95 | (8.94) |
| Tract % age 25+ with some college education* | 28.94 | (7.39) | 29.42 | (7.68) | 29.59 | (7.52) | 28.05 | (7.01) |
| Tract % Hispanic* | 14.62 | (19.32) | 14.51 | (18.71) | 16.78 | (20.91) | 12.43 | (17.56) |
| % of households 5+ not speaking English well* | 4.92 | (7.47) | 4.8 | (7.24) | 5.29 | (7.99) | 4.59 | (6.98) |
| Tract % age 25+ with <12-year education for Whites* | 15.14 | (12.74) | 13.05 | (11.6) | 16.02 | (13.26) | 15.19 | (12.58) |

**Table S2. Simulation scenarios for evaluating the coverage probability of the proposed summary metrics.** The coverage probability of the proposedsummary metric (i.e., $\bar{AUC}_{w}$ for summary discrimination and $\bar{BS}_{w}$for summary accuracy) was assessed in multiple simulation settings by varying landmark intervals (LMIs), prediction windows (w) and censoring rate, as shown in the table below. For example, in generating each simulated dataset under LMI=4, we used a 4-year interval for the landmarks at which we generated longitudinal data, i,e., $S=\left\{ 0,4,8 \right\}$ (Please see **Supplementary Method 3.1.** for detailed methods for simulating a landmark model).

| Scenario | Landmarks (*S*) | Prediction window (*w*) | Censoring Rate (%) |
| --- | --- | --- | --- |
| LMI=2 | 0, 2, 4 years | 2 years | 0, 15, 30, 50 |
| LMI=4 | 0, 4, 8 years | 4 years | 0 |
| LMI=6 | 0, 6, 12 years | 6 years | 0 |

Abbreviations: LMI: landmark interval, *S*: landmarks, *w*: prediction window.

**Table S3. Simulation results of evaluating the coverage probability of the proposed summary metrics.** We assessed the coverage probability of the proposed metric (i.e., summary Brier Score, denoted $\bar{BS}_{w}$ and summary AUC, denoted $\bar{AUC}_{w}$)’s confidence interval under varying sample sizes (*n*=500, 750, 1500), landmark intervals (i.e., LMI 2, 4, and 6 years) and censoring rates (0%, 15%, 30%, 50% at baseline). The coverage probability is defined as the probability that the proposed metric’s confidence interval contains the true metric value (i.e., true $\bar{BS}_{w}$ and $\bar{AUC}_{w}$ value) assumed in each simulated dataset. The data generation process is described in **Supplementary Methods 3.1.** We used a 5% significance level to estimate a 95% confidence interval over 500 simulations, expecting a valid coverage probability to be close to 95%. For example, for *n* = 500, LMI = 2 years (i.e., landmark times of *S* = {0, 1, 2}) and no censoring (0%), the confidence interval of $\bar{AUC}_{w}$ has a coverage rate of 94.81, meaning 94.81% of the time, the estimated confidence interval contained the true value (66.90 in this case). This is illustrated in **Figure S1**.

|  |  |  | *n*=500 | | *n*=750 | | *n*=1500 | |
| --- | --- | --- | --- | --- | --- | --- | --- | --- |
| Censoring Rate (%) | LMI | aTrue value | bMean value | cCP | Mean value | CP | Mean value | CP |
| ***Summary AUC*** | | | | | | | | |
| 0 | 2 | 66.90 | 66.75 | 94.81 | 66.71 | 94.41 | 66.89 | 94.61 |
| 0 | 4 | 68.76 | 68.76 | 95.81 | 68.65 | 95.21 | 68.71 | 95.81 |
| 0 | 6 | 69.47 | 69.39 | 94.41 | 69.44 | 93.41 | 69.48 | 95.81 |
|  |  |  |  |  |  |  |  |  |
| 15 | 2 | 66.90 | 66.88 | 94.41 | 66.73 | 93.21 | 66.95 | 94.61 |
| 30 | 2 | 66.90 | 66.80 | 95.21 | 66.72 | 94.61 | 66.93 | 95.41 |
| 50 | 2 | 66.90 | 66.89 | 95.41 | 66.75 | 93.61 | 66.92 | 94.61 |
| ***Summary Brier Score*** | | | | | | | | |
| 0 | 2 | 0.201 | 0.202 | 95.81 | 0.202 | 96.21 | 0.201 | 96.81 |
| 0 | 4 | 0.217 | 0.218 | 93.61 | 0.218 | 90.82 | 0.217 | 93.01 |
| 0 | 6 | 0.210 | 0.211 | 93.61 | 0.211 | 92.22 | 0.210 | 93.41 |
|  |  |  |  |  |  |  |  |  |
| 15 | 2 | 0.201 | 0.203 | 92.22 | 0.203 | 91.82 | 0.201 | 91.82 |
| 30 | 2 | 0.201 | 0.202 | 94.81 | 0.202 | 95.21 | 0.201 | 95.01 |
| 50 | 2 | 0.201 | 0.202 | 93.61 | 0.202 | 93.41 | 0.201 | 93.81 |

Abbreviations: CP: coverage probability, LMI: landmark interval, *n*: sample size.

Note:

aThe “True value” column shows the true $\bar{BS}_{w}$ and $\bar{AUC}_{w}$ value used in each set of simulations (see **Supplementary Methods 3.2**)**.**

bThe “Mean value” column shows the average $\bar{BS}_{w}$ and $\bar{AUC}_{w}$ over the simulations

cThe “CP” column shows the coverage probability for the proposedsummary metric.

**Table S4. Simulation results of the coverage probability of the traditional time-dependent AUC and time-dependent Brier Score.** Under varying sample sizes (*n*=500, 750, 1500) and landmark intervals (i.e., LMI 2, 4, and 6 years), we assessed the coverage probability of the traditional time-dependent metrics (i.e., time-dependent Brier Score, denoted $BS(s,s+w)$ and time-dependent AUC, denoted $AUC(s,s+w)$)’s confidence interval at each landmark. The data generation process is described in **Supplementary Methods 3.1.** We used a 5% significance level to estimate a 95% confidence interval over 500 simulations, expecting a valid coverage probability to be close to 95%.

|  |  |  | *n*=500 | | *n*=750 | | *n*=1500 | |
| --- | --- | --- | --- | --- | --- | --- | --- | --- |
|  | aLandmark | bTrue value | cMean value | dCP | Mean value | CP | Mean value | CP |
| ***Time-dependent AUC*** | | | | | | | | |
| LMI=2 | $s_{0}=0$ | 67.73 | 67.49 | 94.61 | 67.50 | 96.81 | 67.72 | 92.81 |
|  | $s_{1}=2$ | 66.76 | 66.62 | 95.41 | 66.64 | 95.61 | 66.82 | 95.41 |
|  | $s_{2}=4$ | 66.20 | 66.16 | 92.81 | 65.98 | 94.81 | 66.14 | 96.01 |
|  |  |  |  |  |  |  |  |  |
| LMI=4 | $s_{0}=0$ | 71.32 | 71.08 | 96.01 | 71.20 | 95.21 | 71.23 | 95.81 |
|  | $s_{1}=4$ | 68.52 | 68.63 | 94.81 | 68.52 | 94.81 | 68.55 | 96.21 |
|  | $s_{2}=8$ | 66.46 | 66.58 | 92.61 | 66.23 | 96.21 | 66.34 | 95.01 |
|  |  |  |  |  |  |  |  |  |
| LMI=6 | $s_{0}=0$ | 74.54 | 74.61 | 93.41 | 74.24 | 93.81 | 74.47 | 96.41 |
|  | $s_{1}=6$ | 64.71 | 64.78 | 94.21 | 64.86 | 94.21 | 64.76 | 95.01 |
|  | $s_{2}=12$ | 69.17 | 68.78 | 94.21 | 69.23 | 91.62 | 69.20 | 95.41 |
|  |  |  |  |  |  |  |  |  |
| ***Time-dependent Brier Score*** | | | | | | | | |
| LMI=2 | $s_{0}=0$ | 0.216 | 0.217 | 95.81 | 0.217 | 96.01 | 0.216 | 95.21 |
|  | $s_{1}=2$ | 0.200 | 0.201 | 94.61 | 0.201 | 96.21 | 0.200 | 95.01 |
|  | $s_{2}=4$ | 0.187 | 0.189 | 95.01 | 0.189 | 92.61 | 0.187 | 95.41 |
|  |  |  |  |  |  |  |  |  |
| LMI=4 | $s_{0}=0$ | 0.210 | 0.211 | 96.21 | 0.211 | 96.01 | 0.211 | 95.41 |
|  | $s_{1}=4$ | 0.223 | 0.224 | 95.01 | 0.224 | 93.21 | 0.223 | 96.21 |
|  | $s_{2}=8$ | 0.217 | 0.218 | 95.21 | 0.218 | 95.41 | 0.217 | 96.61 |
|  |  |  |  |  |  |  |  |  |
| LMI=6 | $s_{0}=0$ | 0.177 | 0.177 | 96.61 | 0.178 | 93.81 | 0.177 | 95.41 |
|  | $s_{1}=6$ | 0.230 | 0.232 | 93.81 | 0.231 | 94.61 | 0.230 | 95.21 |
|  | $s_{2}=12$ | 0.222 | 0.225 | 95.01 | 0.223 | 92.02 | 0.223 | 94.81 |
|  |  |  |  |  |  |  |  |  |

Abbreviations: CP: coverage probability, LMI: landmark interval.

Note:

aThe “Landmark” column shows at which landmark the time-dependent metrics are assessed. For example, for LMI=2 and landmark $s_{0}=0$, $BS(0,0+2)$ and $AUC(0,0+2)$ are assessed.

b“True value” column shows the true $BS(s,s+w)$ and $AUC(s,s+w)$ value used in each set of simulations (see **Supplementary Methods 3.2**)**.**

cThe “Mean value” column shows the average $BS(s,s+w)$ and $AUC(s,s+w)$ over the simulations

dThe “CP” column shows the coverage probability for the time-dependent metrics.

**Table S5. Simulation results of the type I error of the comparison test based on the proposed summary metrics.** The type I error rates of the proposedperformance comparison test for summary discrimination (i.e.,$\mathcal{H}_{0}:\Delta\bar{AUC}_{w}=0$ ) or summary predictive accuracy ($\mathcal{H}_{0}:\Delta\bar{BS}_{w}=0$) between two alternative prediction models ($M_{1}$ and $M_{1}^{*}$) are shown below. The simulation setting is described in **Supplementary Method 3.3.** In brief, 500 datasets were simulated and analyzed under the null hypothesis with a sample size of *n*=3000. To each simulated dataset, two alternative dynamic models, $M_{1}$ and $M_{1}^{*}$—which have the same predictive information (i.e., meeting the null hypothesis; see **Supplementary Method 3.3**)—were fit. After fitting the models, we estimated the proposed summary metrics $\bar{AUC}_{w=5}$ and $\bar{BS}_{w=5}$ for each model to compare the difference in these metrics across models. The significance levels of 0.01, 0.025, 0.05, and 0.1 were evaluated along with different censoring rates (0%, 15%, 30%, 50% at baseline).

|  | Censoring rate (%) at baseline | | | |
| --- | --- | --- | --- | --- |
| $\alpha$ | 0 | 15 | 30 | 50 |
|  |  |  |  |  |
| ***Summary Brier score test:*** $\mathcal{H}_{0}:\Delta\bar{BS}_{w}=0$ | | | | | |
| 0.1 | 0.10 | 0.09 | 0.08 | 0.08 |
| 0.05 | 0.05 | 0.05 | 0.04 | 0.03 |
| 0.02 | 0.02 | 0.02 | 0.02 | 0.01 |
| 0.01 | 0.01 | 0.01 | 0.01 | 0.00 |
|  |  |  |  |  |
| ***Summary AUC test:*** $\mathcal{H}_{0}:\Delta\bar{AUC}_{w}=0$ | | | | | |
| 0.1 | 0.10 | 0.09 | 0.10 | 0.09 |
| 0.05 | 0.05 | 0.04 | 0.04 | 0.04 |
| 0.02 | 0.02 | 0.02 | 0.01 | 0.01 |
| 0.01 | 0.01 | 0.01 | 0.00 | 0.00 |
|  |  |  |  |  |

# Supplementary Figures

**Figure S1. Illustrating coverage rates: confidence intervals of the proposed summary AUC across 100 simulations.** Results are shown for the proposedsummary AUC (denoted $\bar{AUC}_{w}$) for a landmark interval (LMI) of 2 years (described in **Table S2**) with *n*=500 over the first 100 simulations. Data generation is described in **Supplementary Methods 3.1.** The coverage rate of the confidence intervals for the proposedsummary AUC (denoted $\bar{AUC}_{w}$) is the proportion of times the confidence intervals contain the true value (dashed line). Confidence intervals that do not contain this value are colored orange. Here, this occurs in 94/100 runs, close to the desired 95%.

**
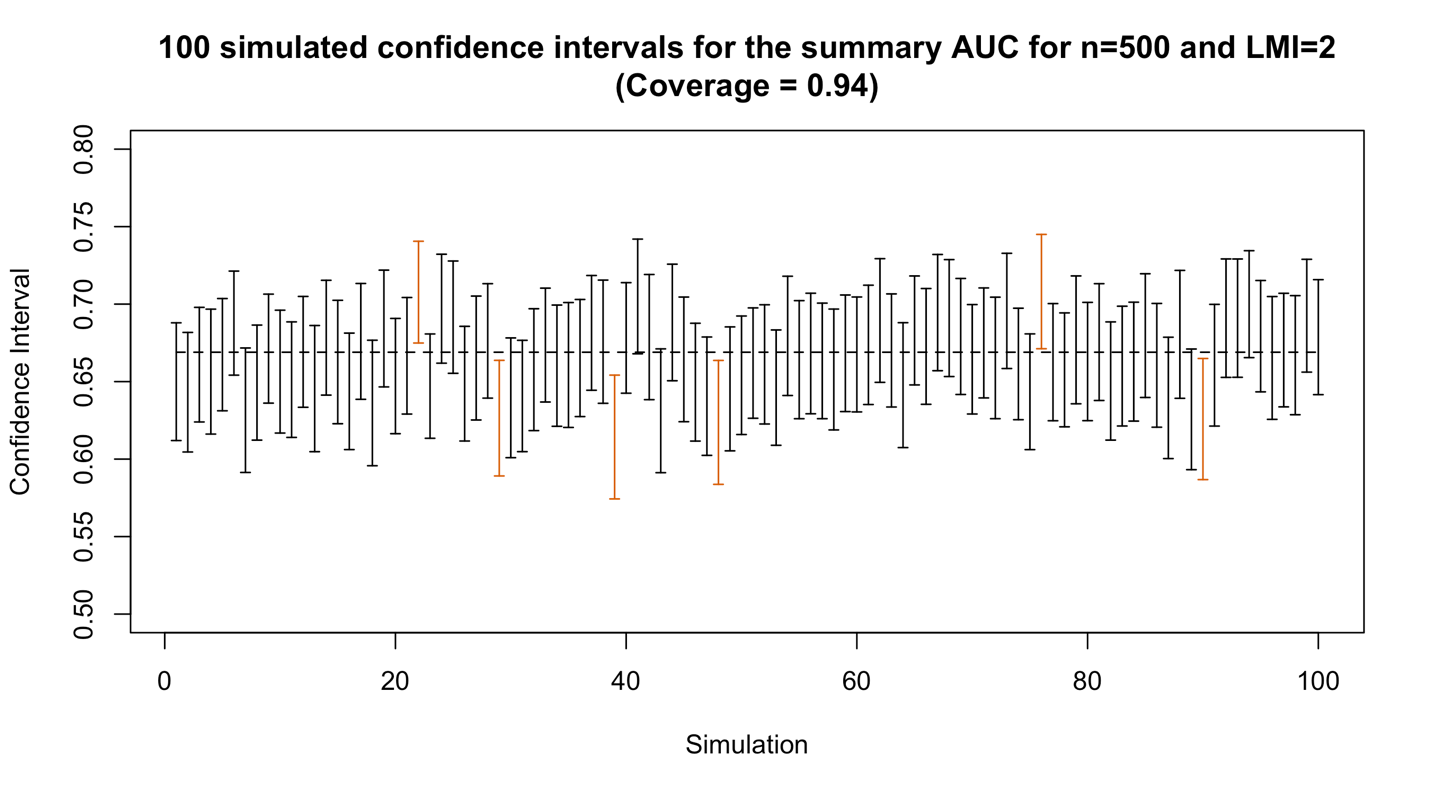
**

Abbreviations: *n*: sample size, LMI: landmark interval.

**Figure S2. Simulation results of the power of the proposed summary performance tests.**

**(A) Results over varying number of landmarks.** The power of the proposedsummary AUC (denoted $\bar{AUC}_{w}$) and Brier Score (denoted $\bar{BS}_{w}$) is shown for a 0.05 significance level for the tests $\mathcal{H}_{0}:\Delta\bar{AUC}_{w}=0$ and $\mathcal{H}_{0}:\Delta\bar{BS}_{w}=0.$ Without the proposed metric, performance comparison relies on testing a set of *k* traditional, landmark-specific time-dependent metrics, adjusted for multiple testing (see **Supplemental Methods 3.4.**), for which we used the Bonferroni method and the Benjamini-Hochberg method to control for a false discovery rate (FDR) of 5%. The data simulation method is further described in **Supplemental Methods 3.4**. The plots show power for varying numbers of landmarks (*k*, shown in the x-axis) for a fixed sample size, *n* and no censoring. Results for $\bar{AUC}_{w}$ and $\bar{BS}_{w}$ are shown in the first and second row, respectively. In each column, the sample size is fixed.


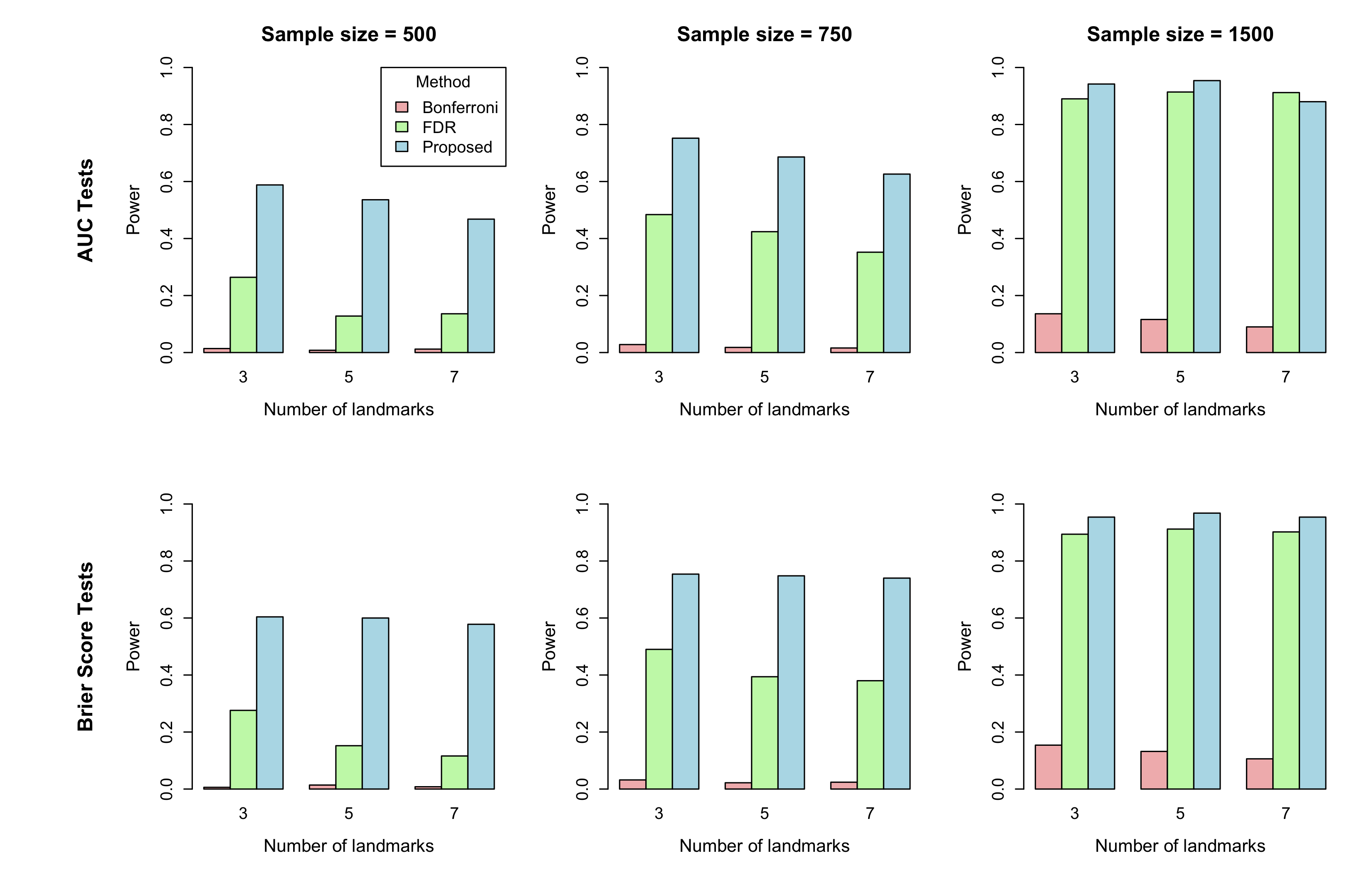


Abbreviations: BS: the proposed summary Brier Score, *k*: number of landmarks, *n*: sample size.

**(B) Results over varying sample size.** The power of the proposedsummary AUC (denoted $\bar{AUC}_{w}$) and Brier Score (denoted $\bar{BS}_{w}$) is shown for a 0.05 significance level for the tests $\mathcal{H}_{0}:\Delta\bar{AUC}_{w}=0$ and $\mathcal{H}_{0}:\Delta\bar{BS}_{w}=0.$ Without the proposed metric, performance comparison relies on testing a set of *k* traditional, landmark-specific time-dependent metrics, adjusted for multiple testing (see **Supplemental Methods 3.4.**), for which we used the Bonferroni method and the Benjamini-Hochberg method to control for a false discovery rate (FDR) of 5%. The data simulation method is further described in **Supplemental Methods 3.4**. The plots show power for varying sample size (*n*, shown in the x-axis) for a fixed number of landmarks, *k* and no censoring. Results for $\bar{AUC}_{w}$ and $\bar{BS}_{w}$ are shown in the first and second row, respectively. In each column, the number of landmarks is fixed.


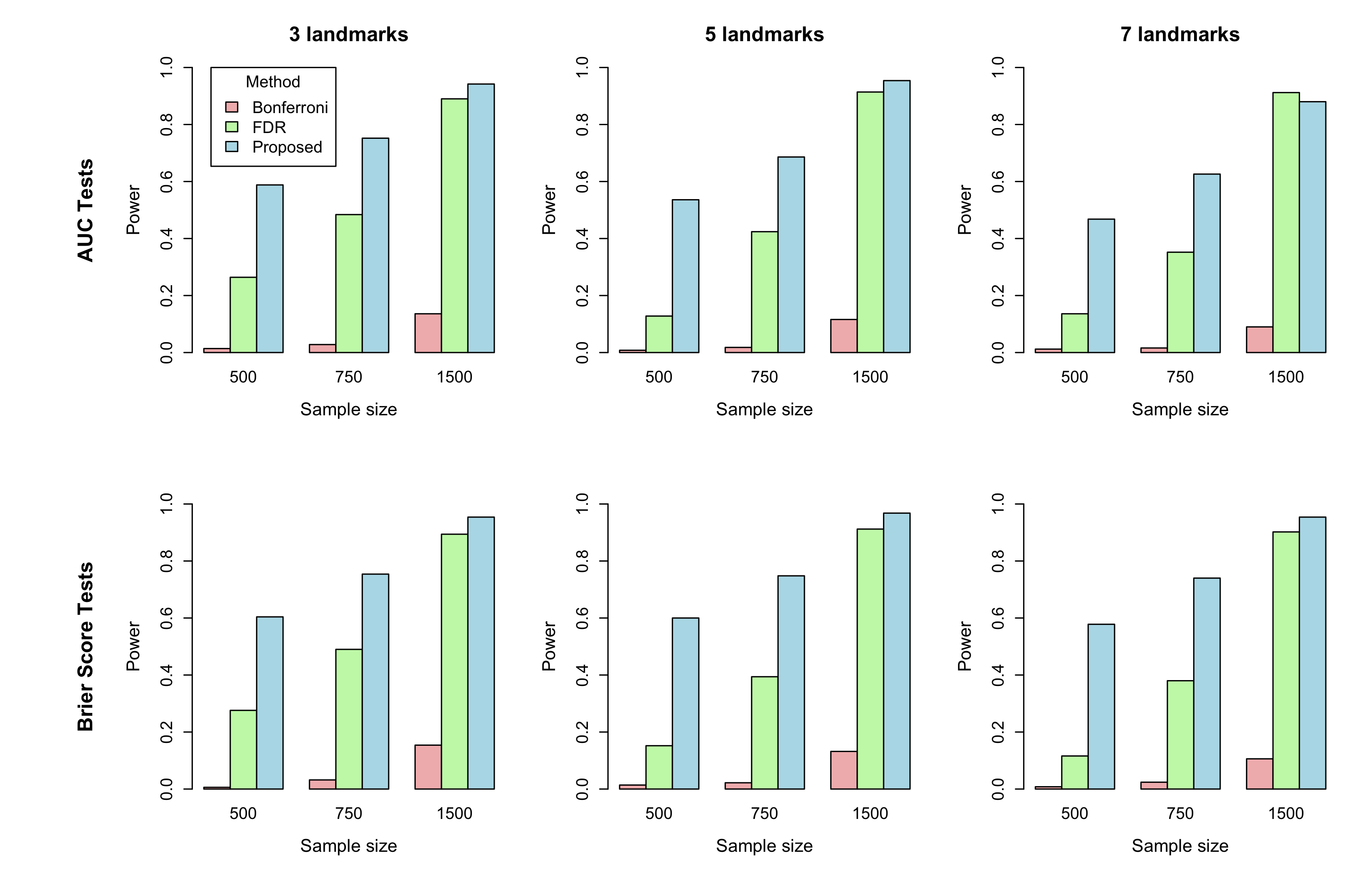


Abbreviations: BS: the proposed summary Brier Score, *k*: number of landmarks, *n*: sample size.

**(C) Results over varying censoring rates.** The power of the proposedsummary AUC (denoted $\bar{AUC}_{w}$) and Brier Score (denoted $\bar{BS}_{w}$) is shown for a 0.05 significance level for the tests $\mathcal{H}_{0}:\Delta\bar{AUC}_{w}=0$ and $\mathcal{H}_{0}:\Delta\bar{BS}_{w}=0.$ Without the proposed metric, performance comparison relies on testing a set of *k* traditional, landmark-specific time-dependent metrics, adjusted for multiple testing (see **Supplemental Methods 3.4.**), for which we used the Bonferroni method and the Benjamini-Hochberg method to control for a false discovery rate (FDR) of 5%. The data simulation method is further described in **Supplemental Methods 3.4**. The plots show power for varying censoring rates (x-axis) for a fixed sample size, *n*. The number of landmarks is always fixed as 3 landmarks. Results for $\bar{AUC}_{w}$ and $\bar{BS}_{w}$ are shown in the first and second row, respectively. In each column, the sample size is fixed.


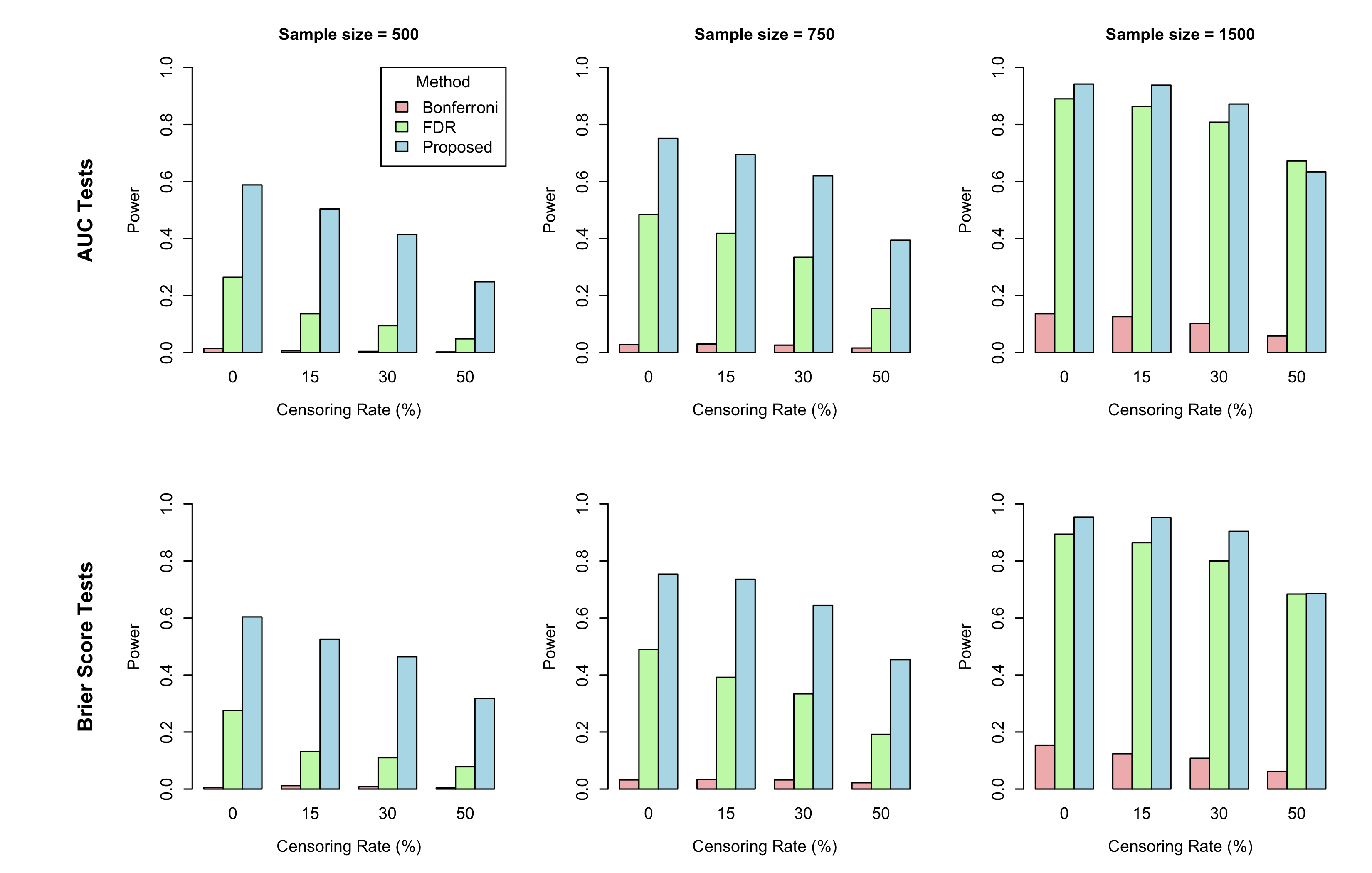


Abbreviations: BS: the proposed summary Brier Score, *n*: sample size.

**Figure S3 Examples of time-dependent effects (coefficient trajectories over time) of three variables using the proposed penLM and alternative (CSC models) for lung cancer mortality analysis.** The time-dependent effects of three selected variables were shown in the following three panels: (i) “percent persons age 25+ with <12 years education” (coded as the percentage, 0-100, in the Census tract), (ii) ever had congenital heart failure (coded 0: no, 1: yes), (iii) on immunotherapy (coded 0: no, 1: yes). The coefficient over time of the penalized landmark (penLM) supermodel (in orange) is compared to landmark-specific cause-specific Cox models (CSC models, in blue). The first two plots show the un-smoothness of the coefficients of the CSC models, leading to difficulty interpreting their meaning. The penLM supermodel avoids this pitfall. The third plot highlights that the CSC models can fail to include relevant covariates due to univariate selection. A patient on immunotherapy had no altered risk in the CSC models, but the penLM supermodel showed a decreasing effect from baseline.


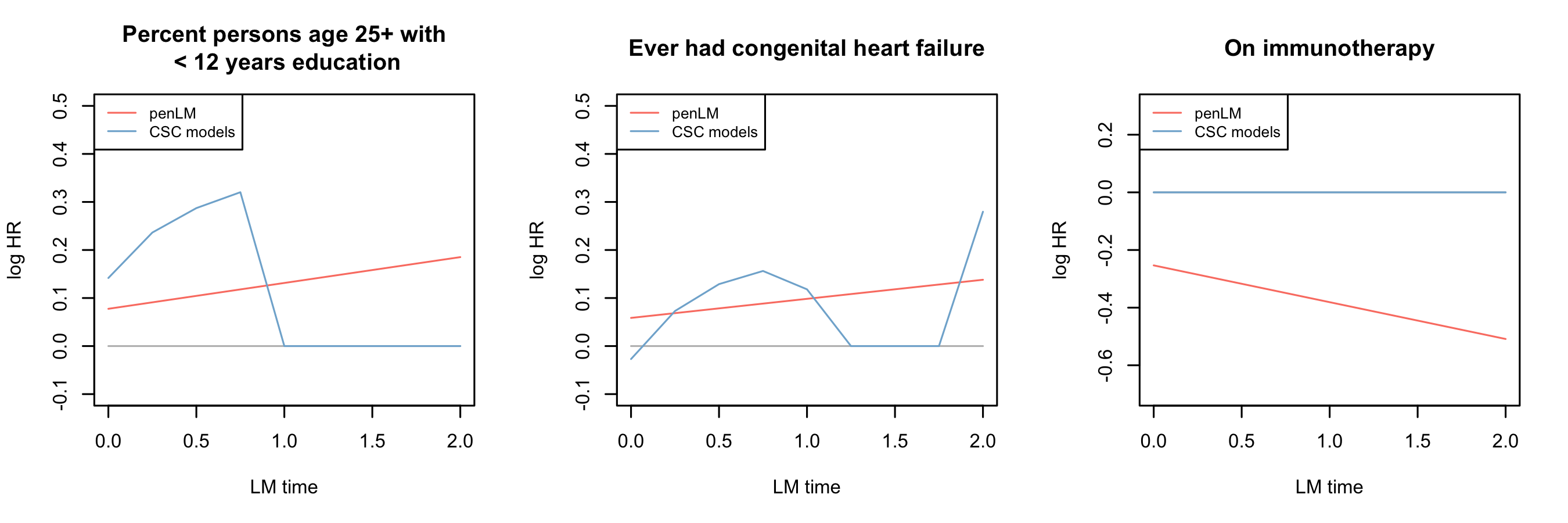


Abbreviations: penLM: penalized landmark supermodel, CSC: cause-specific Cox, logHR: log hazard ratio.

# Supplementary Methods

## **The landmark supermodel**

**1.1. Overview**


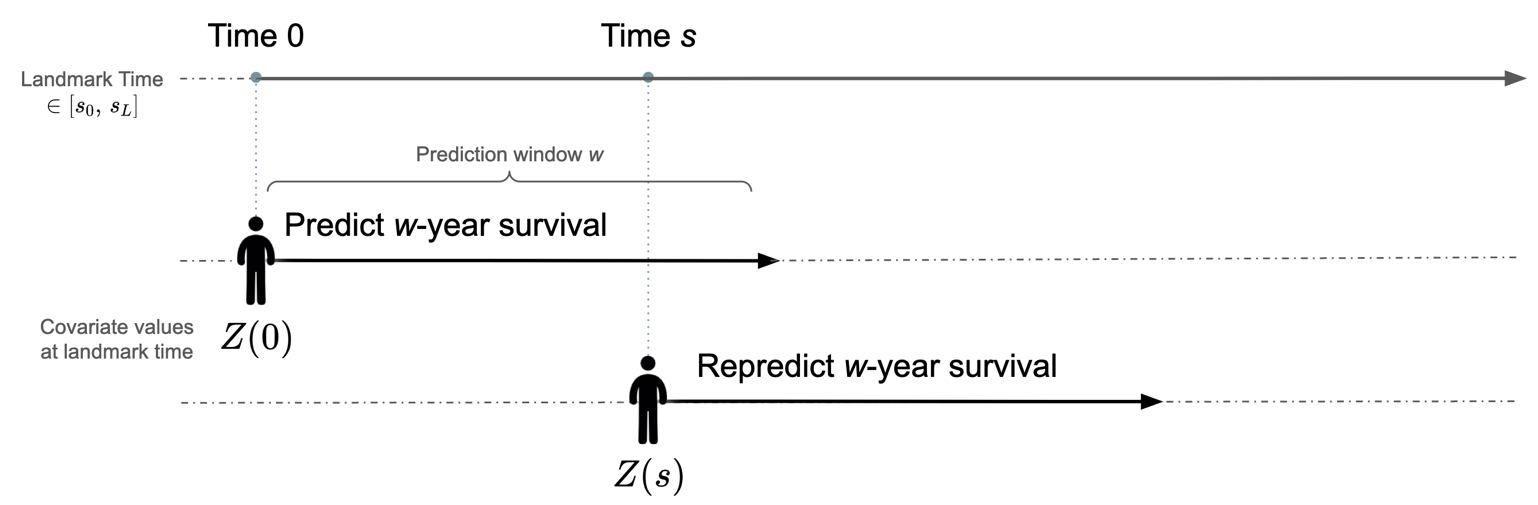
The landmark model for survival data is built on the concept of risk assessment times (i.e., landmarks) that span risk prediction times of interest, using the information on the individuals who survived up to that given time point. In this approach, the dataset of the study cohort is transformed into multiple censored datasets based on a prediction window of interest and the predefined landmark times. A model is fit on the stacked super dataset (i.e., supermodel), and dynamic $w$-year risk prediction is then performed by using the most up-to-date value of a patient's covariate values. Specifically, risk prediction for the next $w$ years is made at baseline (e.g., diagnosis) as well as at a later set of risk assessment times (“landmark prediction times”) after baseline (e.g., at 1, 2, and 3 years after diagnosis), where $w$ is a fixed prediction window.

## **1.2. Landmarking**

Landmarking for risk prediction on survival data is a method for $w$-year dynamic risk prediction where an individual has a personalized risk prediction which is updated as new information is collected among those who survived at a given time for risk assessment. Traditionally, a separate Cox proportional hazards (PH) model is applied to each landmark dataset.1 Predictions can then be made at each landmark time point. The landmark supermodel combines these models by introducing smoothing to permit risk prediction at any landmark.2

Landmarking was first introduced as a concept by Anderson et al. (1983)3, was adapted to dynamic prediction for survival by van Houwelingen (2007)4, and then used in the context of time-dependent covariates by van Houwelingen and Putter (2008)5. It was then conceptualized for competing risks by Nicolaie and van Houwelingen (2012)2. The following framework is explained in further detail in van Houwelingen (2007)4 and Nicolaie and van Houwelingen (2012)2.

## **1.3. Sliding landmark model and extension to competing risks**

Let $w$ be the prediction window of interest. We aim to create a model to estimate $w$-year risk at a landmark time $s$, knowing an individual’s covariates at $s$, $Z(s)$, and conditional on being alive at $s$. To create the landmark model, risk prediction times of interest are first partitioned into different landmarks $\{s_{0},\ldots,s_{L}\}$. The *sliding landmark dataset* is created for each landmark $s$, using only the data of individuals at risk (i.e., not censored or having experienced an event) with administrative censoring applied after $s+w$. For a time-dependent covariate, the dataset contains the most recent covariate value of the patient $Z(s)$, which is considered as a fixed variable.

Suppose that individuals can experience one of $J$ types of failure ('causes'). Competing risk analysis accounts for the probability of other causes of failure, by estimating the probability of a specific event while considering the presence of competing events. Each event (‘cause’) is modelled through a cause-specific hazard.

To create the sliding landmark model, a separate cox model is fit to each dataset by maximizing the Cox partial likelihood to find the parameters $\beta\left( s \right)$: leading to a conditional hazard of the following form:

- Standard survival analysis:

$h\left( t | Z\left( s \right),s \right)=h_{0}\left( t | s \right)\exp\left( Z\left( s \right)\beta\left( s \right) \right)$

$s\leq t\leq s+w$

- Under competing-risk, for cause $j$

$h_{j}\left( t | Z\left( s \right),s \right)=h_{j0}\left( t | s \right)\exp\left( Z\left( s \right)\beta_{j}\left( s \right) \right)$

$s\leq t\leq s+w, 1\leq j\leq J$

In summary, the sliding landmark model consists of different models for each landmark, and predictions can only be made at these predefined landmarks.

## **1.4. The landmark supermodel**

At a high-level, the landmark supermodel introduces smoothing between the separate sliding landmark models, to create one model that provides $w$-year prediction at any $s\in[s_{0},s_{L}]$.

A super dataset is built to create the landmark supermodel:

1. Fix $w$, a prediction horizon/window, and partition risk prediction times of interest into different landmarks $\{s_{0},\ldots,s_{L}\}$.
2. Build a landmark data set for each $s_{i}$ as before (i.e., with left truncation at $s_{i}$, right administrative censoring after $s_{i}+w$, and using the covariate values $Z(s_{i})$)
3. Stack these data sets to create a “super prediction data set.”

A Cox (or cause-specific Cox) model is trained on the super dataset. To account for covariate landmark-varying effects, the regression coefficients depend smoothly on $t_{LM}=s$ (modelled linearly), i.e., $\beta\left( s \right)= \sum_{k} \beta_{k}f_{k}(s)$ for some functions $f_{k}(s)$. The default in our implementation is

$$\beta\left( s \right)= \beta_{0}+ \beta_{1}s+ \beta_{2}s^{2}$$

Note that the regression parameters depend on prediction time, not event time! The baseline hazard alsodepends on $s$, and this can be modelled by: $h_{0}\left( t | s \right)=h_{0}\left( t \right)\exp(\alpha\left( s \right))$. The default in our implementation is

$$\alpha\left( s \right)= \alpha_{0}+\alpha_{1}s$$

Such a model is then fit on the super dataset, which leads to the hazard:

$$h_{j}\left( t | Z\left( s \right),s \right)=h_{j0}\left( t \right)\exp\left( \alpha_{j}\left( s \right)+ \beta_{j}\left( s \right)Z(s) \right), 1\leq j\leq J$$

In summary, the main effects for the landmark time $s$ is modeled by $\alpha(s)$ and the interaction of $s$ with the covariates is modeled by $\beta(s)$. The baseline hazard at time $t$ when predicting from landmark $s$, $h_{j0}\left( t \right)\exp\left( \alpha_{j}\left( s \right) \right)$, is the probability that a person with all zero covariates will experience the event $j$ in the instant *t* if that person survived from *s*. Dependence between entries needs to be accounted for, for example, by using a robust sandwich estimator, as the same patient appears multiple times in the super dataset.

## **Prediction**

Prediction uses the most up-to-date patient covariates. The cumulative hazard is defined as $H\left( s+w | Z\left( s \right),s \right)=\int_{s}^{s+w} h\left( t | Z\left( s \right),s \right)dt$. Under the Cox model (time-to-event data), survival $S$ and cumulative incidence $F$ are as follows:

$$S\left( s+w | Z\left( s \right),s \right)=\exp\left( -\int_{s}^{s+w} h\left( t | Z\left( s \right),s \right)dt \right)$$

$$F\left( s+w | Z\left( s \right),s \right)=1- S\left( s+w | Z\left( s \right),s \right)$$

Under competing risks, all cause-specific hazards are considered, with coefficients from each of the cause-specific Cox models, $\beta_{j}\left( s \right)= \beta_{j0}+ \beta_{j1}s+ \beta_{j2}s^{2}$ and $\alpha_{j}\left( s \right)= \alpha_{j0}+\alpha_{j1}s$. To compute survival, the cumulative cause-specific hazards are summed:

$$S\left( s+w | Z\left( s \right),s \right)=\exp\left( -\int_{s}^{s+w} \sum_{j=1}^{J} h_{j}\left( t | Z\left( s \right),s \right)dt \right)$$

$$=\exp\left( -\sum_{s<t_{i}\leq s+w} \sum_{j=1}^{J} h_{j}\left( t_{i} | Z\left( s \right),s \right) \right)$$

where $t_{i}$ are event times in the original dataset.

Cause-specific cumulative incidence considers the chance of failure from cause $j$ in each interval conditional on surviving to that point:

$$F_{j}\left( s+w | Z\left( s \right),s \right)=\int_{s}^{s+w} h_{j}\left( t | Z\left( s \right),s \right)S\left( t | Z\left( s \right),s \right)dt$$

$$=\sum_{s<t_{i}\leq s+w} h_{j}\left( t_{i} | Z\left( s \right),s \right)S\left( t_{i} | Z\left( s \right),s \right)$$

Note that for the sliding landmark model, $h_{j}\left( t | Z\left( s \right),s \right)=h_{j0}\left( t | s \right)\exp\left( Z\left( s \right)\beta_{j}\left( s \right) \right)$ and for the supermodel, $h_{j}\left( t | Z\left( s \right),s \right)=h_{j0}\left( t \right)\exp\left( \alpha_{j}\left( s \right)+ \beta_{j}\left( s \right)Z(s) \right)$.

## **Penalization (penLM)**

With a large dataset and time-dependent effects, the supermodel has many parameters. We introduce penalization to ensure better generalization while handling much higher dimensionality. A model is fit by maximizing the penalized pseudo-partial likelihood (PPL) of the supermodel$.$

For a single-cause model the unpenalized PPL is given by:

$$ipl^{*}\left( \beta,\alpha\right):=\prod_{i=1}^{n} \prod_{\left\{ s: s\leq T_{i}\leq s+w \right\}} \left( \frac{\exp\left( Z_{i}\left( s \right)^{T}\beta\left( s \right)+\alpha\left( s \right) \right)}{\sum_{\left\{ s: s\leq T_{i}\leq s+w \right\}} \sum_{j\in R\left( T_{i} \right)} \exp\left( Z_{j}\left( s \right)^{T}\beta\left( s \right)+\alpha\left( s \right) \right)} \right)^{\eta_{i}}$$

Where $R(T)$ is the risk set of patients alive at $T$ and $\eta_{i}, T_{i}$ are respectively if the event occurred and time-to-event for patient $i$. When multiple causes/competing risks are present, the PPL factors over the *J* competing events,assuming an independent censoring mechanism.9 This allows for the PPL to be maximized by maximizing individual cause-specific Cox models. The PPL for *J* competing events is given by:

$$ipl^{*}(\boldsymbol{\beta}, \boldsymbol{\alpha})=\prod_{j=1}^{J} ipl^{*}(\beta_{j}, \alpha_{j})$$

Where **­­**and $\boldsymbol{\alpha}=(\alpha_{1},\ldots, \alpha_{J})$, the cause-specific coefficients.

The penalized log PPL for a single-cause model is given by the following equation where the penalty $p\left( \cdot\right)$ can be a LASSO6 (the L1 norm), Ridge7 (the L2 norm), or an elastic net8 (a combination of the two):

$$\log ipl^{*}\left( \beta,\alpha\right)-\lambda p\left( \beta, \alpha\right)$$

$$:=\sum_{i=1}^{n} \log\left( \prod_{\left\{ s: s\leq T_{i}\leq s+w \right\}} \left( \frac{\exp\left( Z_{i}\left( s \right)^{T}\beta\left( s \right)+\alpha\left( s \right) \right)}{\sum_{\left\{ s: s\leq T_{i}\leq s+w \right\}} \sum_{j\in R\left( T_{i} \right)} \exp\left( Z_{j}\left( s \right)^{T}\beta\left( s \right)+\alpha\left( s \right) \right)} \right)^{\eta_{i}} \right)-\lambda p(\beta, \alpha)$$

For competing events, as the PPL factors over the *J* competing events assuming an independent censoring mechanism, the penalized log PPL factors, too:

$$\sum_{j=1}^{J} \left\{ \log\left( ipl^{*}\left( \beta_{j}, \alpha_{j} \right) \right)-\lambda_{j}p\left( \beta_{j}, \alpha_{j} \right) \right\}$$

Where $\lambda_{j}$ is a cause-specific penalty. Penalization is thus essentially performed on each cause-specific Cox model separately, in line with the unpenalized method.

Penalization leads to a trade-off between the model complexity and goodness-of-fit, where the optimal weights $\lambda_{j}$ are chosen via the cross-validated penalized log PPLs.10 The methods used for prediction remain the same using the values for $\beta_{j}, \alpha_{j}$ obtained.

## **Evaluating model performance**

## **Traditional metrics**

Current methods to evaluate dynamic risk prediction models involve evaluating calibration and discrimination at each landmark point individually.

*Dynamic time-dependent AUC*11 measures discrimination. As the landmark model performs $w$-year risk prediction, discrimination between those who incur events before and after $w$-year prediction is of interest. Simply put, it is the percentage of correctly ordered markers when comparing a case (those who incur the event of interest before the window) and a control (those who do not)- – i.e., those who incur the primary event within the window $w$ after prediction and those who do not. More formally, it is defined as the ratio of the estimated probability of observing a pair of a case and a control with ordered markers over the estimated probability of observing a pair with a case and a control. Let the $t$-year risk prediction (marker) of an individual $i$ with covariate information $Z_{i}(s)$ at time $s$ be labeled $M_{i}(s,t)$ and let $M_{i}\left( 0,t \right):=M_{i}$. Their time-to-event is $T_{i}$, censoring time $C_{i}$, $\tilde{T}_{i}=min(T_{i}, C_{i})$, $\eta_{i}$ is the event that $i$ undergoes, $\Delta_{i}$ is whether an individual incurs censoring (where 0 indicates censoring) and $\tilde{\eta}_{i}=\Delta_{i}\eta_{i}$.

Under time-to-event data, time-dependent AUC is defined as:

$$AUC\left( t \right)=P\left( M_{i}\left( 0,t \right)>M_{k}(0,t) \right| T_{i}\leq t, T_{k}>t)$$

The cases $i$ are the individuals who incur an event before $t$, and controls $k$ are those that survive past $t.$ Under competing-risks this becomes,

$$AUC\left( t \right)=P\left( M_{i}\left( 0,t \right)>M_{k}\left( 0,t \right) \right| T_{i}\leq t,\eta_{i}=1,\left\{ T_{k}>t \right\}\mathrm{or}\{T_{k}\leq t, \eta_{k}\neq1\})$$

The cases $i$ are the individuals with the event of interest (for simplicity, written as the first event) before $t$, and the controls $k$ are those that survive past $t$ or incur a competing risk before $t$.

When extending to landmark times, the cases are those that experience the event in $\left[ s,s+w \right]$ and controls survive past them. The time-to-event and competing-risk definitions are:

$$AUC\left( s,t \right)=P\left( M_{i}(s,t)>M_{k}(s,t) \right| {s\leq T}_{i}\leq t,T_{k}>t)$$

$$AUC\left( s,t \right)=P\left( M_{i}\left( s,t \right)>M_{k}\left( s,t \right) \right| {s\leq T}_{i}\leq t,\eta_{i}=1,\left\{ T_{k}>t \right\}\mathrm{or}\{{s\leq T}_{k}\leq t, \eta_{k}\neq1\}) )$$

When performing dynamic risk prediction using landmarking, $AUC\left( s,s+w \right)$ is calculated for each landmark $s$ used in prediction.

It should be noted that the C-index is not appropriate as it assesses the order of the event times and not the order of the event status at the prediction horizon.

$$\boldsymbol{C} = P\left( M_{i}>M_{k} \right|T_{i}<T_{k})$$

*Dynamic time-dependent Brier11* is also provided. The Brier Score summarizes the squared difference between event indicators $\eta_{i}$ and risk estimates $M_{i}.$ Its formulation combines definitions of the expected Brier score for competing risks12, which is the average squared difference between the primary event markers at the end of the prediction horizon and the absolute risk estimates by that time point, and for dynamic prediction13 leading to

$$BS\left( s,t \right)\mathbb{= E}\left[ \left( 1\left\{ s\leq T<s+w, \eta_{i}=1 \right\}-M\left( s,t \right) \right)^{2} \right|T>s]$$

When performing dynamic risk prediction using landmarking, $BS(s,s+w)$ is calculated for each landmark $s$ used in prediction.

*Calibration plots* are important to assess the agreement between predictions and observations in different percentiles of the predicted values. These can be plotted for each of the landmarks used for prediction.

## **Model performance: estimators and inference for traditional metrics**

We briefly summarize the results of Blanche (2015)11 on inverse-probability-of-censoring weighting (IPCW) estimators for $AUC\left( s,t \right)$ and $BS\left( s,t \right)$, building confidence intervals, model performance tests, and some asymptotic results.

***IPCW estimators:*** Building on the previous notation, let $\tilde{D}_{i}\left( s,t \right)=1_{\{s< \tilde{T}_{i}\leq s+t, \tilde{\eta}_{i}=1\}}$ which equals 1 when subject $i$ is known to have experienced the main event within the time interval $(s, s+t]$. Let $\hat{S}_{\tilde{T}}\left( u \right)=\frac{\sum_{i=1}^{n} \boldsymbol{1}_{\{\tilde{T}_{i}>u\}}}{n}$ estimate the probability of observing a subject at risk at $s$. Further, let $\hat{G}(u)$ be the Kaplan-Meier estimator of the survival function of the censoring time at $u$, i.e., $P(C>u)$. For all $u>s$, $\hat{G}\left( u | s \right):= \hat{G}(u)/\hat{G}(s)$ estimates the conditional probability of not being censored at time $u$, conditionally on being uncensored at time $s$.

Now define the weight:

$$\hat{W}_{i}\left( s,t \right)=\frac{1_{\{\tilde{T_{i}}> s+t\}}}{\hat{G}\left( s+t | s \right)}+ \frac{1_{\{s < \tilde{T_{i}} \leq s+t, \tilde{\eta}_{i}\neq0\}}}{\hat{G}\left( \tilde{T}_{i} | s \right)}$$

Then the IPCW estimators are as follows:

$$\hat{AUC}(s,t)=\frac{\sum_{i=1}^{n} \sum_{k=1}^{n} {1_{\{M_{i}\left( s,t \right)>M_{k}(s,t)\}}\tilde{D}}_{i}\left( s,t \right)\left( 1-\tilde{D}_{k}\left( s,t \right) \right)\hat{W}_{i}\left( s,t \right)\hat{W}_{k}\left( s,t \right)}{\sum_{i=1}^{n} \sum_{k=1}^{n} \tilde{D}_{i}\left( s,t \right)\left( 1-\tilde{D}_{k}\left( s,t \right) \right)\hat{W}_{i}\left( s,t \right)\hat{W}_{k}\left( s,t \right)}$$

$$\hat{BS}\left( s,t \right)=\frac{1}{n \hat{S}_{\tilde{T}}\left( s \right)}\sum_{i=1}^{n} \hat{W}_{i}\left( s,t \right)\left( \tilde{D}_{i}\left( s,t \right) - M_{i}\left( s,t \right) \right)^{2}$$

These estimators are model-free in the sense that there is no assumption about the correctness of the specification of the model used for computing $M_{i}(s,t)$.

***Confidence intervals:*** Letting $\theta(s,t)$ denote either $AUC(s,t)$ or $BS(s, t)$ and $\hat{\theta}(s,t)$ the corresponding IPCW estimator, Blanche (2015)11­showed that, assuming that the censoring time $C$ is independent of $(T, \eta, M(\cdot, \cdot)),$ and imposing some simple identifiability constraints on $s$ and $t$,

$$\sqrt{n} \left( \hat{\theta}\left( s,t \right)-\theta(s,t) \right)=\frac{1}{\sqrt{n}}\sum_{i=1}^{n} \gamma^{i}+o_{p}(1)$$

Where $\gamma^{i}:=\gamma\left( i,s,t \right)=IF_{\theta}(T_{i}, \eta_{i}, M_{i}\left( s,t \right), s, t)$, $\mathbb{E}\left[ \gamma^{i} \right]=0$ and $IF_{\theta}(\cdot)$ is the influence function of the estimator, defined in the Appendix A of Blanche (2015)11­. From the decomposition of the estimator in a sum of asymptotically i.i.d. terms, the central limit theorem induces the asymptotic normality of the estimator. By using a plug-in estimator $\hat{IF}_{\theta}(\cdot)$, the variance $\sigma^{2}$ can be consistently estimated by the empirical estimator $\hat{\sigma}_{s,t}$ (defined below), which can be used to construct confidence intervals.

$$\hat{\sigma}_{s,t}=\frac{1}{n}\sum_{i=1}^{n} \hat{IF}_{\theta}\left( \tilde{T_{i}}, \tilde{\eta_{i}}, M_{i}^{\left( 1 \right)}\left( s,t \right), s, t \right)^{2}$$

***Model comparisons:*** Consider two rival models with marker values $M_{i}^{\left( 1 \right)}\left( \cdot, \cdot\right)$ and $M_{i}^{\left( 2 \right)}\left( \cdot, \cdot\right)$, leading to performances $\theta^{\left( 1 \right)}\left( s,t \right)$ and $\theta^{\left( 2 \right)}(s,t)$. Setting $\Delta\theta\left( s,t \right)= \theta^{\left( 1 \right)}\left( s,t \right)- \theta^{\left( 2 \right)}(s,t)$, its’ IPCW estimator $\Delta\hat{\theta}\left( s,t \right)= \hat{\theta}^{\left( 1 \right)}\left( s,t \right)- \hat{\theta}^{\left( 2 \right)}(s,t)$, and the estimated influence function ${\Delta\hat{IF}}_{\theta}\left( \tilde{T_{i}}, \tilde{\eta_{i}}, M_{i}^{\left( 1 \right)}\left( s,t \right),M_{i}^{(2)}\left( s,t \right), s, t \right)=\hat{IF}_{\theta}\left( \tilde{T_{i}}, \tilde{\eta_{i}}, M_{i}^{\left( 1 \right)}\left( s,t \right), s, t \right)-\hat{IF}_{\theta}\left( \tilde{T_{i}}, \tilde{\eta_{i}}, M_{i}^{\left( 2 \right)}\left( s,t \right), s, t \right)$, a test for comparing two predictive performances can be computed. Under $\mathcal{H}_{0}:\Delta\theta\left( s,t \right)=0$, as $n\to\infty$, $\sqrt{n}\left( \Delta\hat{\theta}\left( s,t \right) /\hat{\sigma}_{\Delta,s,t} \right)\mathcal{\to N}(0,1)$ in distribution, where:

$$\hat{\sigma}_{\Delta,s,t}=\frac{1}{n}\sum_{i=1}^{n} {\Delta\hat{IF}}_{\theta}\left( \tilde{T_{i}}, \tilde{\eta_{i}}, M_{i}^{\left( 1 \right)}\left( s,t \right),M_{i}^{(2)}\left( s,t \right), s, t \right)^{2}$$

***Additional asymptotic results:*** As the landmark model considers $t = s+w$ with $w$ a fixed window, we simplify the notation from now on, writing: $\theta\left( s \right):= \theta\left( s,s+w \right), \hat{\theta}\left( s \right):= \hat{\theta}\left( s,s+w \right)$ and $\gamma^{i}\left( s \right):= \gamma\left( i,s,s+w \right)=IF_{\theta}(T_{i}, \eta_{i}, M_{i}\left( s,s+w \right), s, s+w)$.

We end this section by presenting some notation introduced in Blanche (2015)11’sproofs.

1. A consistent U-statistic estimator of $\theta(s)$ is provided, which we denote $U_{n}(s)$.
2. A Hájek projection of $U_{n}\left( s \right)$, which we denote $\pi_{n}\left( s \right)$, is also provided. This is exactly the previously introduced quantity $\pi_{n}\left( s \right)= \frac{1}{\sqrt{n}} \sum_{i=1}^{n} \gamma^{i}(s)$.

Further, the proofs provide two important results:

1. The consistency of $U_{n}$ is sup-controlled:

$$\sup_{s} \left| \sqrt{n} \left( \hat{\theta}\left( s \right)-\theta(s) \right)-U_{n}\left( s \right) \right|=o_{p}(1)$$

2. As is its Hájek projection,

$$\sup_{s} \left| \sqrt{n} \left( \hat{\theta}\left( s \right)-\theta(s) \right)-\pi_{n}(s) \right|=o_{p}(1)$$

## **Model performance: novel summary metrics**

We propose novel model-agnostic metrics (for both discrimination and predictive accuracy) that summarize (average) the traditional time-dependent metrics. As the same individuals are included over different time points, these metrics are correlated over time. We account for the temporal correlation when deriving a confidence interval using an i.i.d. decomposition. Let $\theta(s,s+w)$ be $AUC\left( s,s+w \right)$ or $BS\left( s,s+w \right)$. The proposed model-agnostic summary (average) of the traditional time-dependent metrics over landmarks $s \in$ $\mathcal{S}$ is:

$$\bar{\theta}_{w}=\frac{1}{\mathcal{|S|}} \sum_{s\in\mathcal{S}} \theta(s,s+w)$$

*Lemma 1. Assuming the censoring time is independent of the events, event times and marker-generating process, there is an i.i.d. decomposition:*

$$\sqrt{n} \left( \hat{\boldsymbol{\theta}}\boldsymbol{-\theta} \right)=\frac{1}{\sqrt{n}}\sum_{i=1}^{n} \boldsymbol{\gamma}^{i}+o_{p}(1)$$

*Where we fix $w$, set* $\theta(s)$ as $AUC\left( s,s+w \right)$ or $BS\left( s,s+w \right)$, *define the vectors $\boldsymbol{\theta}=\left\{ \theta\left( s \right) \right\}_{s\in\mathcal{S}}=\left\{ \theta\left( s,s+w \right) \right\}_{s\in\mathcal{S}}, \hat{\boldsymbol{\theta}}$ its IPCW estimate, and $\boldsymbol{\gamma}^{i}=\left\{ \gamma^{i}\left( s \right) \right\}_{s\in\mathcal{S}}$ with $\gamma^{i}\left( s \right)=IF_{\theta}(T_{i}, \eta_{i}, M_{i}\left( s,s+w \right), s, s+w)$.*

*Proof.*

| We first note that the Hájek projection of the landmark-specific metrics (2) generalizes to higher dimension. Blanche showed that there exists an i.i.d. decomposition of the landmark-specific estimators of the form $\pi_{n}(s)= \frac{1}{\sqrt{n}} \sum_{i=1}^{n} \gamma^{i}(s)$ where $\gamma^{i}(s)=IF_{\theta}\left( T_{i}, \eta_{i}, M_{i}\left( s,s+w \right), s, s+w \right).$ However, it is further true that $\gamma^{i}(s)=IF_{\theta}\left( T_{i}, \eta_{i}, M_{i}\left( \cdot,\cdot\right), s, s+w \right)$ (i.e., knowing the marker $M(s,s+w)$ or the marker-generating process $M(\cdot, \cdot)$, does not change the estimate). Thus, the Hájek projection of the vector $\boldsymbol{U}_{n}=\left\{ U_{n}(s) \right\}_{s\in\mathcal{S}}$ is the same as the entry-wise projections. Specifically, by setting $\boldsymbol{\gamma}^{i}=\left\{ \gamma^{i}\left( s \right) \right\}_{s\in\mathcal{S}}$, we have that: $\boldsymbol{\pi}_{n}:=\frac{1}{\sqrt{n}} \sum_{i=1}^{n} \boldsymbol{\gamma}^{i}$ is the Hájek projection of $\boldsymbol{U}_{n}$ From the decomposition of $\boldsymbol{\pi}_{n}$ as a sum of asymptotically i.i.d. variables, the central limit theorem ensures asymptotic normality for some covariance matrix $\boldsymbol{\Sigma}$, $\boldsymbol{\pi}_{n}= \frac{1}{\sqrt{n}} \sum_{i=1}^{n} \boldsymbol{\gamma}^{i} \underset{\to}{\mathcal{D}}\mathcal{N}(0, \boldsymbol{\Sigma})$ Since (4) gives us sup control, i.e., $\sup_{s} \left\vert\sqrt{n} \left( \hat{\theta}\left( s \right)-\theta\left( s \right) \right)-\pi_{n}(s) \right\vert=o_{p}(1)$ and $\boldsymbol{\pi}_{n}$ is indeed the Hájek projection, we have the result of the lemma, and that: $\sqrt{n} \left( \hat{\boldsymbol{\theta}}\boldsymbol{-\theta} \right)\underset{\to}{\mathcal{D}}\mathcal{N}(0, \boldsymbol{\Sigma})$ |
| --- |

***Confidence intervals:*** Using the same plug-in estimator as Blanche (2015)11­*,* $\hat{IF}_{\theta}(\cdot)$, and setting ${\hat{\boldsymbol{\gamma}}}^{i}={{\hat{\{IF}}_{\theta}\left( \tilde{T_{i}}, \tilde{\eta_{i}}, , M_{i}\left( \cdot,\cdot\right), s, s+w \right)\}}_{s\in\mathcal{S}}$ the covariance matrix $\boldsymbol{\Sigma}$ can be consistently estimated by:

$$\hat{\boldsymbol{\Sigma}}\mathbf{=}\frac{1}{n-1}\sum_{i=1}^{n} {{\hat{\boldsymbol{\gamma}}}^{i}({\hat{\boldsymbol{\gamma}}}^{i})}^{T}$$

Set $m=\left| \mathcal{S} \right|$, the number of landmarks, then define $g\left( \boldsymbol{\theta} \right)=\frac{1}{m}\sum_{s\mathcal{\in S}} \theta\left( s \right)$ which has gradient $\nabla g(\boldsymbol{\theta})=\frac{1}{m}1_{m}$ where $1_{m}$ is the vector of ones of dimension $m$. The summary metric is exactly $\bar{\theta}_{w}=g(\boldsymbol{\theta})$. Thus, using the delta method, we can obtain a $(1-\alpha)$-level confidence interval for the summary metric $\bar{\theta}_{w}$ as follows.

$$\left\{ \bar{\theta}_{w}\pm z_{1-\frac{\alpha}{2}}\frac{\hat{\sigma}_{w}}{\sqrt{n}} \right\}$$

where $\hat{\sigma}_{w}^{2}= \nabla g\left( \hat{\boldsymbol{\theta}} \right)^{T} \hat{\boldsymbol{\Sigma}} \nabla g\left( \hat{\boldsymbol{\theta}} \right)$ and where $z_{1-\alpha/2}$ is the $1-\alpha/2$ quantile of the univariate standard normal distribution.

***Inference for model comparisons:*** Similarly to Blanche (2015)11­, consider two rival models with marker values $M_{i}^{\left( 1 \right)}\left( \cdot, \cdot\right)$ and $M_{i}^{\left( 2 \right)}\left( \cdot, \cdot\right)$, leading to performances $\boldsymbol{\theta}^{\left( 1 \right)}$ and $\boldsymbol{\theta}^{\left( 2 \right)}$ with difference $\Delta\hat{\boldsymbol{\theta}}\boldsymbol{=}\boldsymbol{\theta}^{\left( 1 \right)}- \boldsymbol{\theta}^{\left( 2 \right)}$. Then define $\Delta{\hat{\boldsymbol{\gamma}}}^{i}= {{\hat{\{IF}}_{\theta}\left( \tilde{T_{i}}, \tilde{\eta_{i}}, M_{i}^{(1)}\left( \cdot,\cdot\right), s, s+w \right)\}}_{s\in\mathcal{S}}- {{\hat{\{IF}}_{\theta}\left( \tilde{T_{i}}, \tilde{\eta_{i}}, M_{i}^{(2)}\left( \cdot,\cdot\right), s, s+w \right)\}}_{s\in\mathcal{S}}$.A confidence interval for the comparison of their summary performance $\Delta\bar{\theta}_{w}=g\left( \Delta\boldsymbol{\theta} \right)$ can be constructed,

$$\left\{ {\Delta\bar{\theta}}_{w}\pm z_{1-\frac{\alpha}{2}}\frac{\hat{\sigma}_{\Delta, w}}{\sqrt{n}} \right\}$$

Where $\hat{\sigma}_{\Delta, w}= \nabla g\left( \Delta\hat{\boldsymbol{\theta}} \right)^{T} {\hat{\boldsymbol{\Sigma}}}_{\Delta} \nabla g\left( \Delta\hat{\boldsymbol{\theta}} \right)$ and ${\hat{\boldsymbol{\Sigma}}}_{\Delta}\mathbf{=}\frac{1}{n-1}\sum_{i=1}^{n} {\Delta{\hat{\boldsymbol{\gamma}}}^{i}(\Delta{\hat{\boldsymbol{\gamma}}}^{i})}^{T}.$ We can also test the null hypothesis $\{\mathcal{H}_{0}: \Delta\bar{\theta}_{w}=0\}$ that the two markers are equivalent. Under $\mathcal{H}_{0}$, as $n\to\infty$, $\sqrt{n}\left( \Delta\bar{\theta}_{w} /\hat{\sigma}_{\Delta,w} \right)\mathcal{\to N}(0,1)$.

## **Simulation Methods**

## **Simulating a landmark dataset**

We generate data from a simple landmark model,14 which satisfies the bilateral relationship between predictors and residual survival outcome. Their data generation algorithm is available on GitHub

(<https://github.com/liwh0904/Compare_JM_and_LM>, last accessed 01/04/2024).

To briefly summarize the algorithm, the data is generated according to the model:

$\lambda\left( t | s, Z\left( s \right) \right)=\lambda_{0}\left( t | s \right)\exp\left( \beta\left( s \right)^{T}Z\left( s \right) \right)$,

Where $\lambda\left( t | s, Z\left( s \right) \right)$ is a hazard that depends on $\lambda_{0}\left( t | s \right)$, the baseline hazard at time $t$ given the landmark $s$, the covariates at landmark *s*, $Z\left( s \right)$, and their coefficients at landmark *s*, $\beta\left( s \right)$. This model implies a relationship between the exponentiated linear predictors $\theta\left( s \right):= \exp\left( \beta\left( s \right)^{T}Z\left( s \right) \right)$ and residual survival outcome $\tilde{T}\left( s \right)=T-s$ where $T$ is the time-to-event.

The user-specified components of the algorithm include the (i) distribution of the exponential linear predictor,$\theta\left( s \right),$ at each landmark, i.e., $f\left( \theta\left( s \right), s \right), \forall s\geq0$ and (ii) the baseline cumulative hazard $\Lambda_{0}\left( t | s=0 \right)=\int_{0}^{t} \lambda_{0}\left( t | s=0 \right)dt$ (note that for $s>0, \Lambda_{0}\left( t | s \right)=\int_{s}^{t} \lambda_{0}\left( t | s \right)dt$ is implied).

Given $\Lambda_{0}\left( t | s=0 \right)$ and $f\left( \theta\left( s \right), s \right), \forall s\geq0$, the time-to-events for individuals are first generated (which gives the residual survival times $\tilde{T}\left( s \right)$). Then, the exponentiated linear predictors, $\theta\left( s \right)$, are generated for any given landmark times. These relationships are written out explicitly by Li et al.14 Once $\theta\left( s \right)$ is generated, we can simulate any $Z(s)$ that satisfy the linear constraints $\theta\left( s \right)= \exp\left( \beta\left( s \right)^{T}Z\left( s \right) \right)$ with pre-specified regression coefficient functions $\beta(s)$.

In our simulations, we set $\Lambda_{0}\left( t | s=0 \right)=\left( 0.6t \right)^{0.9}$ and the distribution of $\theta\left( s \right)$ at each landmark time $s$ to a beta distribution with the following pdf, as used by Li et al.14

$$f(\theta(s), s) \propto x^{a\left( s \right)-1}\left( 1-x \right)^{b\left( s \right)-1}, \mathrm{with} a\left( s \right)=2-0.02s \mathrm{and} b\left( s \right)=2.5+0.05s,$$

We then generate three causal variables, $X_{1},X_{2},$ and $X_{3}$, i.e., $Z\left( s \right)=\left( X_{1},X_{2},X_{3}\left( s \right) \right)$. Among them, $X_{1}$ and $X_{2}$ are static variables (or fixed variables), i.e., they do not change their values over time. These variables were generated from $X_{1}\sim\mathrm{Bernoulli}\left( 0.5 \right), X_{2}\mathcal{\sim N}\left( {0,2}^{2} \right)$. The third variable, $X_{3},$ was set to change its value by landmark time $s$ (i.e., time-varying variable) to satisfy $\theta\left( s \right)= \exp\left( \beta\left( s \right)^{T}Z\left( s \right) \right)$ using constant coefficients over time, $\beta\left( s \right)=(1.2, 0.6, 1.1)$.

We generated this longitudinal data of $X_{3}$ and $\theta\left( s \right)= \exp\left( \beta\left( s \right)^{T}Z\left( s \right) \right)$ across three landmarks and varied the landmark intervals (LMIs) between the landmarks to examine how the landmark interval potentially impacts the performance of the proposed methods. For instance, with a 2-year landmark interval, longitudinal data was generated across landmarks 0, 2, and 4 years, denoted as *S* = {0,2,4}. Similarly, we applied landmark intervals of 4 years (*S* = {0,4,8}) and 6 years (*S* = {0,6,12}). Censoring was added using an independently exponential distribution with rate 4.6, 12, and 31 to respectively censor approximately 50%, 30%, and 15% of subjects. These simulation scenarios are detailed in **Table S1.** It is noted that competing events were not included in this simulation as the evaluation of time-dependent metrics was shown to be robust under these conditions in the prior study.11 Specifically, results were shown to hold for the case where approximately 20% of individuals incurred the main event, 10% a competing event, 20% were censored, and 50% experienced event-free survival at *s+w.*

We also note that simulating each landmark dataset does not require any assumption on a prediction window (*w*). Instead, this prediction window is used to fit the landmark model using the given dataset. Assuming complete knowledge of the underlying model, one would estimate the model parameters using the same landmark interval as in the data generation process and the appropriate prediction window, i.e., set the prediction window as the landmark interval. In our simulations, this is how we fit the supermodel. However, we note that the metrics are model-agnostic and so a mis-specified model should not affect results.

- 1. **Evaluating coverage probability**

We evaluated the new summary metric's finite sample behavior by first assessing the coverage probability of the proposed metric’s confidence interval, i.e., the probability that the confidence interval contains the true metric value (i.e., true $\bar{BS}_{w}$ and $\bar{AUC}_{w}$ values assumed in a simulated dataset). For a 95% confidence interval, this should happen ~95% of the time.

We assessed the coverage probability of the proposed summary AUC, $\bar{AUC}_{w}$, or Brier Score, $\bar{BS}_{w}$, under simulation settings by varying (i) sample size (*n* = 500, 750, 1500), (ii) landmark interval (LMI = 2, 4, 6 years), and (iii) censoring rate (0%, 15%, 30%, 50%). For each scenario, we simulated 500 different training and testing datasets (i.e. 500 random seeds) with a sample size *n* for each of the training and testing datasets. For each seed in each scenario, we fit the landmark supermodel to the training dataset using the same parameters as data generation (i.e., using the same landmarks and a prediction window equal to the landmark interval). Predictions are made on the test dataset at each landmark. A set of time-dependent AUCs and Brier scores were calculated at each landmark, which were then used to estimate the proposed summary metrics $\bar{AUC}_{w}$ and $\bar{BS}_{w}$ and their 95% confidence intervals. For each scenario, we counted the fraction of the datasets where the estimated confidence interval included the “true” $\bar{BS}_{w}$ or $\bar{AUC}_{w}$ value (described in the next paragraph). This is illustrated in **Figure S1** over 100 simulations for n=500 and LMI=2.

We calculated the values of the true summary AUC, $\bar{AUC}_{w}$, or Brier Score, $\bar{BS}_{w}$ for each simulation scenario based on prior study.11 For each scenario, we simulated 1500 different training and test datasets (i.e., 1500 different random seeds) with a sample size of 3000 for each of training and test dataset. For each seed, we fit a landmark supermodel to the training dataset using the same parameters as data generation. Predictions are made on the test dataset from which $\bar{BS}_{w}$ and $\bar{AUC}_{w}$ are estimated. As the metrics are unbiased, the true value for $\bar{BS}_{w}$ and $\bar{AUC}_{w}$ are considered the average across the random seeds for each landmark interval.

The same methods are used for assessing the time-dependent metrics.

- 1. **Evaluating type I error of the proposed performance comparison test**

We then evaluate the type 1 error of the proposed performance comparison tests (i.e., testing for $\mathcal{H}_{0}:\Delta\bar{AUC}_{w}=0$ and $\mathcal{H}_{0}:\Delta\bar{BS}_{w}=0$) between two dynamic prediction models. This assesses how often we reject the null hypothesis (i.e., reject $\mathcal{H}_{0}$) when there is no difference between the two models. We used the data generation method described in **Supplementary Method 3.1.,** with the following modifications**.** Each dataset was generated with four (instead of three) causal variables, $X_{1},{X_{2}, X}_{2*},$ and $X_{3}$, where $X_{2*}$ is a newly added variable, which was generated from the same distribution as $X_{2}$, i.e., $X_{2*}\sim\mathcal{N}\left( {0,2}^{2} \right)$. The regression coefficients for these four factors in simulating each landmark model was set to $\beta\left( s \right)=(1.2, 0.6, 0.6, 1.1)$.

We simulated 500 training and test datasets (i.e., 500 random seeds) using a sample size of *n*=3000 for each dataset and landmarks at *S* = {0, 2, 4} (i.e., a landmark interval [LMI] of 2 in **Table S1**). Censoring rate at baseline (0%, 15%, 30%, 50%) was varied.

Two equivalent landmark supermodels ($M_{1}$ and $M_{1}^{*}$) were fit to each simulated dataset with a prediction window of *w* = LMI = 2. Model $M_{1}$ used three causal variables, $X_{1},X_{2},X_{3}$, whereas $M_{1}^{*}$ used $X_{1},X_{2*},X_{3}$. Thus, the predictive performance of the two models, $M_{1}$ and $M_{1}^{*}$, is equivalent because $X_{2}$ and $X_{2*}$ were generated identically and hence the models have the same predictive information (i.e., meet the null hypothesis).

After fitting the models, predictions were made on the test dataset. A set of time-dependent AUCs and Brier scores were calculated at each time point of 0, 2, and 4 years, which were then used to estimate the proposed summary metrics $\bar{AUC}_{w=2}$ and $\bar{BS}_{w=2}$, for each model. These metrics were used for testing if the model performance difference $\mathcal{H}_{0}:\Delta\bar{AUC}_{w}=0$ or $\mathcal{H}_{0}:\Delta\bar{BS}_{w}=0$. We used significance levels of $\alpha$ = {0.01, 0.02, 0.05, 0.1} and thus counted the proportion of tests such that the P-value was less than or equal to $\alpha$.

## **Evaluating the power of the proposed performance comparison test**

Similarly, we evaluated the power of the proposed performance comparison test (i.e., testing for $\mathcal{H}_{0}:\Delta\bar{AUC}_{w}=0$ or $\mathcal{H}_{0}:\Delta\bar{BS}_{w}=0$) between two different dynamic prediction models. Here, we assess how often we reject the null hypothesis given that it is untrue, under varying alternative hypotheses and scenarios. In particular, we varied (i) the sample size (*n* = 500, 750, 1500), (ii) the number of landmarks (*k* = 3, 5, 7) and (iii) the censoring rate (0%, 15%, 30%, 50%) in generating each landmark dataset. In each scenario, we used the landmark interval (LMI) size set to 2. For example, for *k* = 3, the landmarks at which we generate longitudinal data are *S* = {0, 2, 4}. Similarly, for *k* = 5 and 7, the landmarks are *S* = {0, 2, 4, 6, 8} and *S =* {0, 2, 4, 6, 8, 10, 12}, respectively.

We used the simulation method described in **Supplementary Method 3.1.**,with the following modifications**.** Each dataset was generated with four causal variables, $X_{1},{X_{2}, X}_{2*},$ and $X_{3}$, as in **Supplementary Method 3.2**. The regression coefficients for these four factors in simulating each landmark model was set to $\beta\left( s \right)=(1.2, 0.6, 0.6, 1.1)$.

We simulated 500 training and test datasets (i.e., 500 random seeds) with of sample size *n* for each dataset. Two alternative landmark supermodels ($M_{1}$ and $M_{2}$) were fit to each simulated dataset with a prediction window of *w* = LMI = 2 to be tested for a performance comparison (i.e., $\mathcal{H}_{0}:\Delta\bar{AUC}_{w}=0$ and $\mathcal{H}_{0}:\Delta\bar{BS}_{w}=0$). Model $M_{1}$ was fit as described in **Supplementary Method 3.3.**, using only three of the four causal factors, $X_{1},$ $X_{2},$ and $X_{3}$. Model $M_{2}$ was fit using all four causal variables, thus imposing better predictive performance for $M_{2}$ vs. $M_{1}$.

After fitting the models, predictions were made on the test dataset. We calculated the time-dependent AUC for each model for each landmark time point in $S=\{s_{1}, s_{2},\ldots,s_{k}\}$ and used the resulting vector of time-dependent AUCs, i.e., $\{AUC(s_{1}, s_{1}+w), AUC(s_{2}, s_{2}+w)\ldots, AUC(s_{k},s_{k}+w)\}$ to calculate the proposed summary metric, a single value, $\bar{AUC}_{w}$, for each model. The metric $\bar{AUC}_{w}$ from each model was then used for testing $\mathcal{H}_{0}:\Delta\bar{AUC}_{w}=0$, in which we expect the difference in $\bar{AUC}_{w}$ between $M_{1}$ and $M_{2}$ is statistically different from zero, if the given test is fully powered. We used a significance level of 0.05 and thus counted the proportion of tests with P-value <= 0.05. Analogous methods were used for testing $\mathcal{H}_{0}:\Delta\bar{BS}_{w}=0$.

Further, we examined how the power of the proposed test potentially compares to those based on existing methods (i.e., traditional time-dependent AUCs or Brier scores), if any. In the absence of the proposed metric that summarizes multiple time-dependent AUCs (or Brier scores) into a single value, one of the most straightforward ways of comparing the predictive performance of two models is to compare a time-dependent AUC (or time-dependent Brier score) at each landmark time separately, i.e., at times $s_{1}, s_{2},\ldots,s_{k}$. This will lead to *k* tests, thus inducing multiple comparisons, where the overall statistical significance level should be adjusted accordingly, either by controlling the family-wise error rate or false discovery rate.15, 16

We used the following methods to compare the power of the tests based on traditional time-dependent AUCs vs. the proposed test. In each simulated dataset under a given landmark set, $S=\{s_{1}, s_{2},\ldots,s_{k}\}$, we consider the vector of time-dependent AUCs, i.e., $\{AUC\left( s_{1}, s_{1}+w \right), AUC\left( s_{2}, s_{2}+w \right),\ldots, AUC(s_{k},s_{k}+w)\}$ for each of $M_{1}$ and $M_{2}$. For each $s$, we conducted a test, $\mathcal{H}_{0}:\Delta AUC\left( s, s+w \right)=0$, that $M_{1}$and $M_{2}$ are equivalent (as proposed by Blanche et al.11). In evaluating the power of the tests based on these traditional time-dependent AUCs with a 5% significance level, we used the two multiple testing correction methods, the Bonferroni correction,15 and controlling for a false discovery rate of 5% (“FDR”) with the Benjamini-Hochberg procedure.16 In particular, we counted the fraction of the simulated datasets (500 random seeds) that yielded at least one of the *k* number of tests with a P-value $\leq\alpha^{*}=0.05/k$ under the Bonferroni method and a q-value ≤ 0.05 when controlling for a false discovery rate of 5%. Analogous methods were used for evaluating the power of the tests based on time-dependent Brier scores.

## **Methods for lung cancer mortality prediction using integrated data sources**

***Study Cohort:*** The study cohort comprised patients diagnosed with lung cancer (ICD10: C34) between 2007 and 2017 in the SEER registries, who were continuously enrolled in Medicare Part D and Medicare Advantage programs, and who completed at least one MHOS survey. **Figure 2** shows a flowchart of the study cohort selection. For MHOS survey data, we considered data collected within 1 year before lung cancer diagnosis as baseline if no data is available at the year of lung cancer diagnosis; data collected after lung cancer diagnosis year was considered time-varying information. For the U.S. Census data, we considered the most recent census track-level information before or at diagnosis as baseline. Census information after lung cancer diagnosis was considered time-varying information. Randomly selected 70% of patients (*n*=3269) were used for model fitting (i.e., training), and the remaining 30% (*n*=1401) were used for testing. Results on evaluating the predictive performance across different models were reported on this held-out test set of 30%.

***Imputation:*** Given that each data source’s missing rates and mechanisms vary, we conducted imputation in each dataset before integrating them. The SEER data (collected at the time of lung cancer diagnosis) was imputed using fully conditional specification (FCS) 17. The MHOS data was imputed using the FCS-GLMM method for longitudinal data 18. Due to the absence of specific methods for categorical and ordered categorical variables within 2-level patient data, where patient characteristics exhibit variability, we resorted to standard imputation, excluding considerations of the clustered effect.

# *Comparative method to penLM: Distinct CSC models*

We fit separate CSC models at each landmark (upon lung cancer diagnosis, 1-, and 2-years following diagnosis) using patients survived at each landmark (i.e., all with lung cancer diagnosis, 1-year survivors, and 2-year survivors). The features of these separate CSC models at each landmark are selected from a univariate analysis at each landmark (i.e., variables with P-values $\leq$ 0.05 for the main cause) to prevent overfitting in the absence of an implementation of the penalized CSC model.

# 4. References

1. van Houwelingen, H. and H. Putter, *Dynamic prediction in clinical survival analysis*. 2011: CRC Press.

2. Nicolaie, M.A., et al., *Dynamic prediction by landmarking in competing risks.* Stat Med, 2013. **32**(12): p. 2031-47.

3. Anderson, J.R., K.C. Cain, and R.D. Gelber, *Analysis of survival by tumor response.* J Clin Oncol, 1983. **1**(11): p. 710-9.

4. Van Houwelingen, H.C., *Dynamic prediction by landmarking in event history analysis.* Scandinavian Journal of Statistics, 2007. **34**(1): p. 70-85.

5. van Houwelingen, H.C. and H. Putter, *Dynamic predicting by landmarking as an alternative for multi-state modeling: an application to acute lymphoid leukemia data.* Lifetime Data Anal, 2008. **14**(4): p. 447-63.

6. Tibshirani, R., *Regression Shrinkage and Selection Via the Lasso.* Journal of the Royal Statistical Society: Series B (Methodological), 2018. **58**(1): p. 267-288.

7. Hoerl, A.E. and R.W. Kennard, *Ridge Regression: Biased Estimation for Nonorthogonal Problems.* Technometrics, 2000. **42**(1): p. 80-86.

8. Zou, H. and T. Hastie, *Regularization and Variable Selection Via the Elastic Net.* Journal of the Royal Statistical Society Series B: Statistical Methodology, 2005. **67**(2): p. 301-320.

9. Prentice, R.L., et al., *The Analysis of Failure Times in the Presence of Competing Risks.* Biometrics, 1978. **34**(4): p. 541-554.

10. Verweij, P.J. and H.C. Van Houwelingen, *Cross-validation in survival analysis.* Stat Med, 1993. **12**(24): p. 2305-14.

11. Blanche, P., et al., *Quantifying and comparing dynamic predictive accuracy of joint models for longitudinal marker and time-to-event in presence of censoring and competing risks.* Biometrics, 2015. **71**(1): p. 102-113.

12. Schoop, R., et al., *Quantifying the predictive accuracy of time-to-event models in the presence of competing risks.* Biom J, 2011. **53**(1): p. 88-112.

13. Schoop, R., E. Graf, and M. Schumacher, *Quantifying the predictive performance of prognostic models for censored survival data with time-dependent covariates.* Biometrics, 2008. **64**(2): p. 603-10.

14. Li, W., L. Li, and B.C. Astor, *A comparison of two approaches to dynamic prediction: Joint modeling and landmark modeling.* Statistics in Medicine, 2023. **42**(13): p. 2101-2115.

15. Dunn, O.J., *Multiple Comparisons among Means.* Journal of the American Statistical Association, 1961. **56**(293): p. 52-64.

16. Benjamini, Y. and Y. Hochberg, *Controlling the False Discovery Rate: A Practical and Powerful Approach to Multiple Testing.* Journal of the Royal Statistical Society. Series B (Methodological), 1995. **57**(1): p. 289-300.

17. van Buuren, S. and K. Groothuis-Oudshoorn, *mice: Multivariate Imputation by Chained Equations in R.* Journal of Statistical Software, 2011. **45**(3): p. 1 - 67.

18. Cao, Y., et al., *Review and evaluation of imputation methods for multivariate longitudinal data with mixed-type incomplete variables.* Statistics in Medicine, 2022. **41**(30): p. 5844-5876.
